# MFRP is a molecular hub that organizes the apical membrane of RPE cells by engaging in interactions with specific proteins and lipids

**DOI:** 10.1073/pnas.2425523122

**Published:** 2025-04-18

**Authors:** Aleksander Tworak, Roman Smidak, Carolline Rodrigues Menezes, Samuel W. Du, Susie Suh, Elliot H. Choi, Sanae S. Imanishi, Zhiqian Dong, Dominik Lewandowski, Kristen E. Fong, Gabriela Grigorean, Antonio F. M. Pinto, Qianlan Xu, Dorota Skowronska-Krawczyk, Seth Blackshaw, Yoshikazu Imanishi, Krzysztof Palczewski

**Affiliations:** ^a^Department of Ophthalmology, Gavin Herbert Eye Institute, University of California, Irvine, CA 92697; ^b^Department of Physiology and Biophysics, University of California, Irvine, CA 92697; ^c^Department of Chemistry, University of California, Irvine, CA 92697; ^d^Department of Molecular Biology and Biochemistry, University of California, Irvine, CA 92697; ^e^Department of Ophthalmology, and Stark Neurosciences Research Institute, Indiana University School of Medicine, Indianapolis, IN 46202; ^f^Proteomics Core Facility, Genome Center, University of California, Davis, CA 95616; ^g^Clayton Foundation Laboratories for Peptide Biology, Salk Institute for Biological Studies, La Jolla, CA 92037; ^h^Department of Ophthalmology, Johns Hopkins University, Baltimore, MD 21205; ^i^Department of Neurology, Institute for Cell Engineering, Kavli Neuroscience Discovery Institute, Johns Hopkins University, Baltimore, MD 21205; ^j^Solomon H. Snyder Department of Neuroscience, Johns Hopkins University, Baltimore, MD 21205

**Keywords:** retina, RPE, MFRP, vision, lipids

## Abstract

Membrane frizzled-related protein (MFRP), present in the retinal pigment epithelium (RPE), is an integral membrane protein essential for ocular development and the normal physiology of the retina. Mutations in MFRP are associated with autosomal recessive nonsyndromic nanophthalmos, leading to severe hyperopia and early-onset retinitis pigmentosa. While several preclinical gene-augmentation and gene-editing trials hold promise for future therapies aimed at stopping degeneration and restoring retinal function, the molecular mechanisms involved in MFRP biology are still not well understood. Here, we studied the biochemical properties of MFRP and the molecular consequences of its loss of function in the retinal degeneration 6 (rd6) mouse model. Using transcriptomic and lipidomic approaches, we observed that accumulation of docosahexaenoic acid (DHA) constitutes a primary defect in the MFRP-deficient RPE. In biochemical assays, we showed that MFRP undergoes extensive glycosylation, and it preferentially binds lipids of several classes, including phosphatidylserine and phosphatidylinositol-4-phosphate; as well as binding to several transmembrane proteins, notably adiponectin receptor 1 (ADIPOR1) and inward rectifier potassium channel 13 (KCNJ13). Moreover, MFRP determines the subcellular localization of ADIPOR1 and KCNJ13 in the RPE in vivo. This feature is altered by MFRP deficiency and can be restored by gene-therapy approaches. Overall, our observations suggest that MFRP constitutes an important interaction hub within the apical membrane of RPE cells, coordinating protein trafficking and subcellular localization within the RPE, and lipid homeostasis within the entire retina.

## Significance

Mutations in the membrane frizzled-related protein (MFRP) gene are associated with disorders affecting ocular development and vision. However, the role of MFRP in eye biology remains unclear. Our study on the biochemical properties of MFRP and the detailed ocular characterization of a MFRP-knock-out mouse strain broadens our understanding of the protein’s molecular function. Additionally, we demonstrate that gene-therapy intervention can restore some of the molecular features that are lost due to MFRP deficiency in the eye. This observation supports efforts to develop treatments that target MFRP, using genetic approaches.

**Fig. 1.**
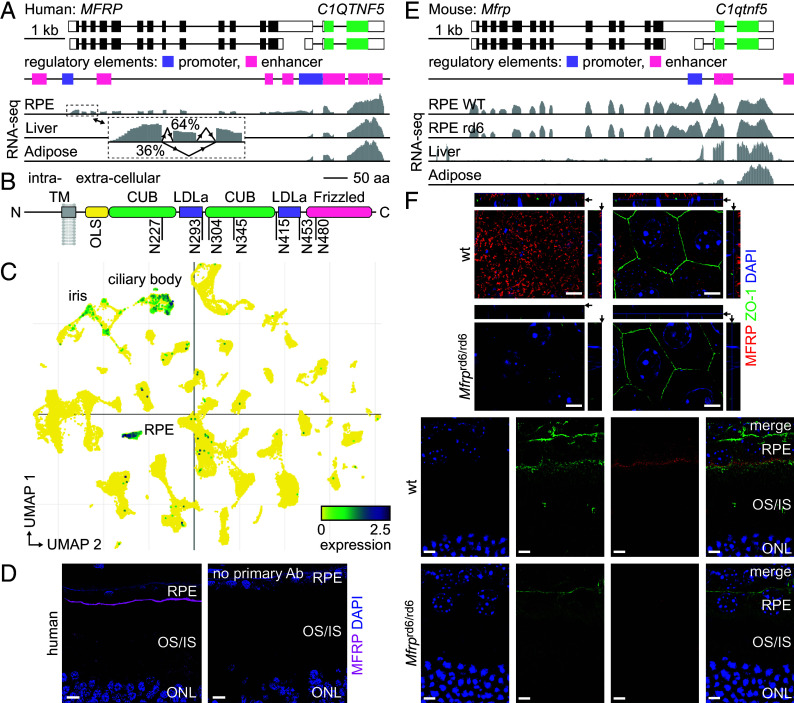
Molecular features of MFRP. (*A*) Gene structures of human *MFRP* and *C1QTNF5,* with tracks indicating location of putative promoter-like and proximal enhancer-like signatures; and RNA-seq read coverage in different tissue types. The inset shows the frequency of *MFRP-*exon-2 alternative splicing events in the RPE. (*B*) Human MFRP domain structure and location of 7 potential N-glycosylation sites and 13 predicted O-glycosylation sites, forming an O-linked sugar (OLS) domain. (*C*) UMAP plot showing MFRP expression level in various human ocular cell types. Significant MFRP expression was observed in the RPE, iris, and ciliary body cells. (*D*) IHC images of cryosections from a human eye. The *Left* section was stained to visualize MFRP and nuclei (DAPI); the *Right* panel shows the staining control, without the primary anti-MFRP antibody. MFRP localizes specifically to the apical membrane of the RPE cells. (Scale bar, 10 µm.) (*E*) Gene structures of mouse *Mfrp* and *C1qtnf5,* with tracks indicating location of putative promoter-like and proximal enhancer-like signatures; and RNA-seq read coverage in different tissue types. (*F*) IHC images of RPE flatmounts (*Top*) and retinal cryosections (*Bottom*) from WT and MFRP-knock-out (retinal degeneration 6, rd6) animals. MFRP forms distinct clusters on the apical membranes of RPE cells. For spatial reference, the tight junction component zonula occludens-1 (ZO-1) was costained on flatmounts; and glucose transporter 1 (GLUT1), which is abundant in both apical and basal RPE membranes, was costained on cryosections. DAPI was used for nuclear staining. (Scale bar, 5 µm.)

Membrane frizzled-related protein (MFRP) has long been recognized as essential for ocular development and normal physiology of the retina (1, 2). Mutations in *MFRP* are associated with autosomal recessive nonsyndromic nanophthalmos, leading to severe hyperopia and early-onset retinitis pigmentosa, frequently accompanied by foveoschisis and optic-nerve-head drusen (3). While several preclinical gene-augmentation (4–8) and gene-editing (9) trials hold promise for future therapies to stop degeneration and restore retinal function, the molecular mechanisms underlying MFRP biology remain largely undefined. The *MFRP* gene encodes a single-pass type-II membrane protein with a short and largely unstructured N-terminal cytoplasmic domain, and a complex extracellular C-terminus harboring two CUB domains, two low-density lipoprotein-receptor domains (LDLa), and a Frizzled domain (Fz) (Fig. 1 *A* and *B*). It is expressed in the retinal pigment epithelium (RPE) and the ciliary epithelium of human and mouse eyes (Fig. 1*C*) (10, 11), and in parts of the brain (12). In the RPE, MFRP localizes predominantly to the apical membrane (Fig. 1*D*), where its absence or dysfunction affects the structure of microvilli interdigitating the outer segments (OS) of the photoreceptors (13, 14). An evolutionarily conserved feature of the *MFRP* gene locus is its proximity to *C1QTNF5,* which encodes complement C1q tumor necrosis factor-related protein 5, a member of the adiponectin superfamily of secretory proteins (Fig. 1 *A* and *E*). Both genes were observed to be transcribed in the form of a single bicistronic mRNA (1), however, an independent functional-promoter region of the *C1QTNF5* gene also has been identified in the human genome (15). MFRP and C1QTNF5 were shown to colocalize in the RPE, and to bind to each other in vitro (10, 16, 17). However, MFRP loss does not affect C1QTNF5 distribution in the retina (13), and mutations in each of the two genes lead to distinct ocular phenotypes, rendering the biological significance of MFRP–C1QTNF5 complex formation unclear. In addition, a functional relationship has been identified between MFRP and adiponectin receptor 1 (ADIPOR1), a ceramidase known for its role in glucose and lipid metabolism. In mice, knock-outs of *MFRP* and *ADIPOR1* lead to similar retinal-disease characteristics, which in certain aspects are affected by an epistatic interaction between the two genes (18). On the molecular level, loss of MFRP leads to the loss of ADIPOR1 from the apical membrane of the RPE cells (19), and mutations in each of the two genes show similar defects in the enrichment and distribution of several important lipids in the retina, including docosahexaenoic acid (DHA, 22:6 n-3) and other polyunsaturated fatty acids (PUFAs) (20). While both C1QTNF5 and adiponectin can induce the phosphorylation of AMP-activated protein kinase (AMPK), regulating lipid and glucose metabolism, only the latter exerts its function through ADIPOR1 binding (21–23). Thus, the molecular details of MFRP function, and of a functional relationship involving MFRP and ADIPOR1, remain to be elucidated.

## Results

### MFRP Undergoes Extensive N- and O-Glycosylation.

Seeking better understanding of the molecular properties of MFRP, we performed in silico analysis of the genetic loci of human and mouse *MFRP* and *C1QTNF5*. In line with human experimental data (15), both human and mouse *C1QTNF5* genes possess putative dedicated promoter sequences, annotated by the ENCODE project (24). Overlay of RNA sequencing (RNA-seq) outputs from the GTEx database (25) showed significant differences between *MFRP* and *C1QTNF5* transcription levels across different tissues (RPE, liver, adipose), overall suggesting independent regulation of the expression of these two genes despite their bicistronic-transcript locale (Fig. 1 *A* and *E*). Notably, in the human RNA-seq data from donor eyes with no known ocular disease, we noticed the occurrence of a significant alternative splicing event, where exon 2 is skipped at a frequency of about 36% (Fig. 1*A*). The resulting transcript would lead to a frameshift that changes the MFRP open-reading frame from codon 19 and markedly shortens its product to just 101 amino acid residues.

Considering the predominantly extracellular topology of MFRP (Fig. 1*B*), we next investigated its glycosylation status. An in silico analysis identified 7 putative N-glycosylation sequons conserved in mammalian MFRP extracellular domains, 5 of which were predicted to undergo sugar modification with high probability (*SI Appendix*, Fig. S1 *A* and *B* and Table S1). In addition, a common feature of mammalian MFRP is a stretch of 13 to 18 (depending on the species) O-linked sugar (OLS) sites (likely O-glycosylation) that are distributed along a 35 to 50-amino acid sequence immediately following the transmembrane domain. This feature is reminiscent of the OLS domain found in extracellular portions of several other membrane proteins (*SI Appendix*, Fig. S1*A* and Table S2) (26). To validate these predictions, we subjected the isolated membrane fractions from native bovine RPE to enzymatic deglycosylation. Removal of either N- or O-linked glycans resulted in mobility shifts of the MFRP band in denaturing-gel electrophoresis, indicating loss of molecular mass (*SI Appendix*, Fig. S1*B*). Not unexpectedly, the largest shift (~25 kDa) was observed in the presence of N-glycanase, suggesting a high cumulative molecular weight of the N-linked carbohydrates. The combined activity of N-glycanase and four enzymes that together catalyze removal of most of the common O-linked carbohydrates led to an additional mobility shift corresponding to an additional loss of ~3 kDa. Considering that OLSs are usually significantly less massive than their N-linked counterparts, this result indicates the presence of multiple O-glycosyl moieties and supports the conclusion that mammalian MFRP has an OLS domain.

### Retinal Degeneration Caused by Loss of MFRP Function does not Affect Rod Phototransduction.

To study the role of MFRP in eye biology, we utilized the retinal-degeneration-6 (rd6, *Mfrp^rd6^*) mouse model, which displays many features in common with human ocular disease, including retinal degeneration and nanophthalmos (1, 6, 27). The loss of MFRP from the rd6 RPE (Fig. 1*F*) leads to slowly progressive thinning of the outer nuclear layer (ONL) accompanied by early-onset subretinal autofluorescent spots (*SI Appendix*, Fig. S2). The structural degeneration correlates with progressive deterioration of visual function, as measured by electroretinography (ERG, a- and b-wave amplitudes at 1 mo of age). MFRP loss in the RPE primarily affects the scotopic responses, indicating greater vulnerability of the rods than the cones to the MFRP dysfunction (*SI Appendix*, Fig. S3 *A*–*C*), as reported previously (13). However, the kinetics of the rod response, analyzed by comparing the leading edge of normalized individual a-waveforms, appeared identical for wild type (WT) and rd6 animals at 1 mo of age, suggesting that rod phototransduction is not affected by the initial degenerative processes (*SI Appendix*, Fig. S3*D*). In contrast, comparison of superimposed normalized waveforms from 6-mo WT and rd6 mice showed noticeably slower kinetics in the rd6 strain, suggesting that MFRP deficiency impairs phototransduction in the remaining rod OS at later stages of the degeneration (*SI Appendix*, Fig. S3*E*).

### Loss of MFRP Function in the RPE Leads to Downregulation of Retinoid and Lipid Metabolic Pathways.

**Fig. 2.**
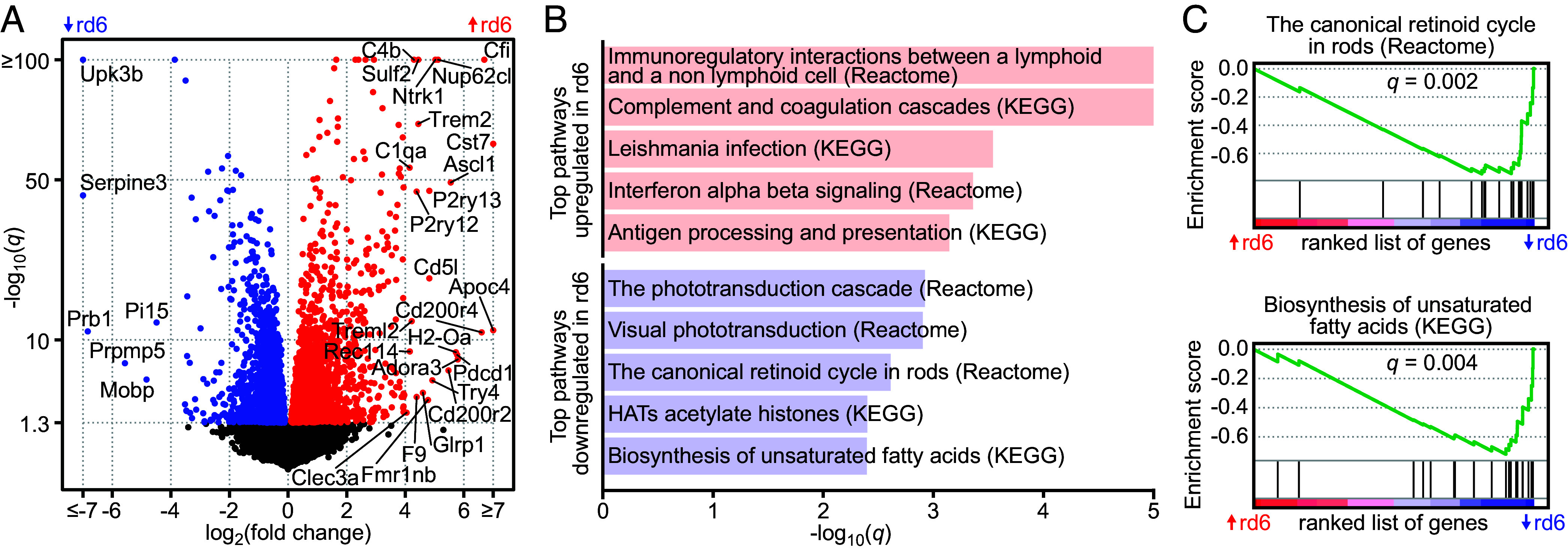
Transcriptomic footprint of the loss of MFRP in the RPE cells. (*A*) Volcano plot illustrating the significantly upregulated (red) and downregulated (blue) genes (*q* < 0.05) in the rd6 RPE, compared to WT cells. Genes with the highest observed fold change are labeled. (*B*) Top scoring significantly up- and down-regulated biological pathways (KEGG and Reactome databases) in the rd6 RPE cells. (*C*) GSEA enrichment plots for the Canonical Retinoid Cycle (Reactome), and the Biosynthesis of Unsaturated Fatty Acids (KEGG) pathways. Distribution of the analyzed pathway genes in the ranked list of all quantified genes is indicated by the black bars on the x axis. Genes were ranked according to their differential expression level and variability, where red and blue indicate up- and downregulation, respectively, in the rd6 RPE.

To gain insight into the molecular roles of the MFRP in eye biology, we compared the RPE transcriptomes of 1-mo WT and rd6 mice. We achieved reliable quantification of 21,279 transcripts, with 4.7 and 2.3% of them exhibiting significant (≥1.75-fold, *q* < 0.05) up- and down-regulation, respectively, in the RPE of the rd6 mice, compared to control conditions (Fig. 2*A*). Further characterization of the transcriptomic data through gene-set enrichment analysis (GSEA) revealed that most relevant gene-set-phenotype associations corresponded to two major categories: gene ontology (GO)-term biological processes, or biological pathways. In both categories, we observed indications of a widespread activation of humoral and complement immune responses in the RPE cells upon the loss of MFRP. Several complement cascade genes (*Cfi*, *F9*, *C4b*, *C1qa*) ranked as the most differentially expressed genes (>16-fold difference) (Fig. 2*A*), and the complement and coagulation cascade was one of top upregulated pathways in the rd6 RPE (Fig. 2*B*). Notably, complement dysregulation contributes to the pathophysiology of both wet and dry forms of age-related macular degeneration (AMD). Pathways involved in visual-chromophore regeneration and in biosynthesis of unsaturated fatty acids displayed the most significant downregulation upon loss of MFRP (Fig. 2*C*). These pathways enable RPE cells to supply the retina with two major molecules vital for photoreceptor health and function: 11-*cis*-retinal and DHA, respectively.

Taking a broader look at genes related to lipid metabolism, which are prominently expressed in the RPE, we observed significant downregulation of two major enzymes specifically involved in DHA processing: major facilitator superfamily domain containing 2A (MFSD2A, fourfold down), a DHA transporter (28); and long-chain acyl-coenzyme A (CoA) synthetase 6 (ACSL6, 1.85-fold down), which was shown to facilitate di-DHA phospholipid biosynthesis (29) (*SI Appendix*, Table S3). The highest upregulation was observed for the cytochrome c oxidase subunit 8B (COX8B, 5.3-fold up), an enzyme of the mitochondrial respiratory chain, indicative of increased mitochondrial biogenesis and lipid metabolism. Additional upregulated genes included inhibitor of DNA binding protein 2 (ID2, 2.5-fold up), a transcription factor involved in the oxidative-stress response in RPE cells (30); and thrombospondin 1 (THBS1, 2.35-fold up), an antiangiogenic and anti-inflammatory extracellular protein that also contributes to the function of the RPE’s blood–retina barrier (BRB) (31).

### MFRP Loss of Function Results in Slower Visual-Cycle Kinetics.

**Fig. 3.**
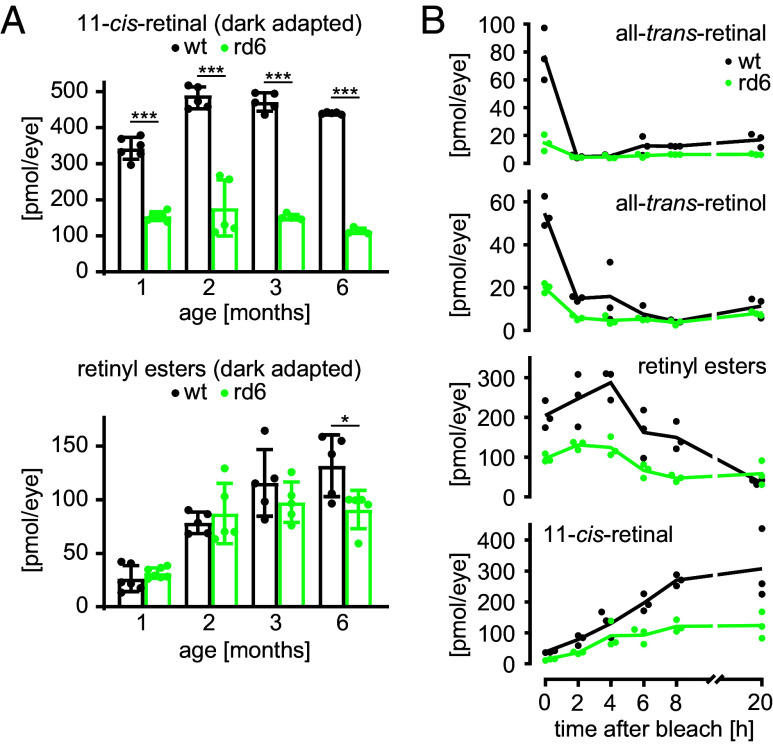
Retinoid profile and visual pigment regeneration kinetics in rd6 mice. (*A*) Analysis of the contents of 11-*cis*-retinal and retinyl esters in the eyes of fully dark-adapted rd6 and WT mice of different ages: 1-, 2-, 3-, and 6-mo. Bars show mean ± SD of n ≥ 5 independent replicates. Significant differences are indicated as follows: ****P < 0.0001, *P < 0.05, two-way ANOVA with the Holm–Šídák post hoc test. (*B*) Accumulation kinetics of all-*trans*-retinal, all-*trans*-retinol, retinyl esters, and 11-*cis*-retinal, 0, 2, 4, 6, 8, and 20 h after a light-bleaching event in the eyes from 1-mo rd6 and WT mice. Bars show mean ± SD of n = 3 independent replicates.

Our transcriptomic analysis showed an approximately twofold decrease in the expression of two major RPE enzymes involved in the classical pathway of 11-*cis*-retinal regeneration, RPE65 and LRAT. To follow up on this observation, we examined the effects on the functionality of the visual cycle. Dark-adapted rd6 animals of different ages (1-, 2-, 3-, and 6-mo) showed consistent, >2-fold decreases in 11-*cis*-retinal levels compared to age-matched controls (Fig. 3*A*). In contrast, the levels of retinyl esters under dark-adapted conditions did not differ significantly between rd6 and WT animals, until they reached 6 mo old. The observation that young rd6 animals retain fully functional phototransduction yet permanently lack a significant portion of 11-*cis*-retinal prompted us to further investigate the kinetics of their visual cycle. Accordingly, we measured the accumulation of all-*trans*-retinal, all-*trans*-retinol, retinyl esters, and 11-*cis*-retinal during the recovery in darkness (up to 20 h) from a light bleaching event (Fig. 3*B*). Immediately after bleaching (0 h) we observed an expected >90% drop in 11-*cis*-retinal levels in the eyes of both rd6 and WT mice, compared to the respective dark-adapted levels. Subsequently, 11-*cis*-retinal started to accumulate; however, replenishment occurred at a ~2-fold-slower rate in the rd6 mice, consistent with their decreased levels of RPE65, the rate-limiting enzyme of the visual cycle. The interim accumulation of retinyl esters peaked earlier in the rd6 mice compared to WT, despite the downregulation of LRAT in the mutant animals. Overall, the data suggest that the visual cycle remains functional in rd6 animals, albeit with its output attenuated by downregulation of major pathway components and the resultant decreased pool of retinoids.

### DHA Accumulation Constitutes a Primary Defect in the MFRP-Deficient RPE.

**Fig. 4.**
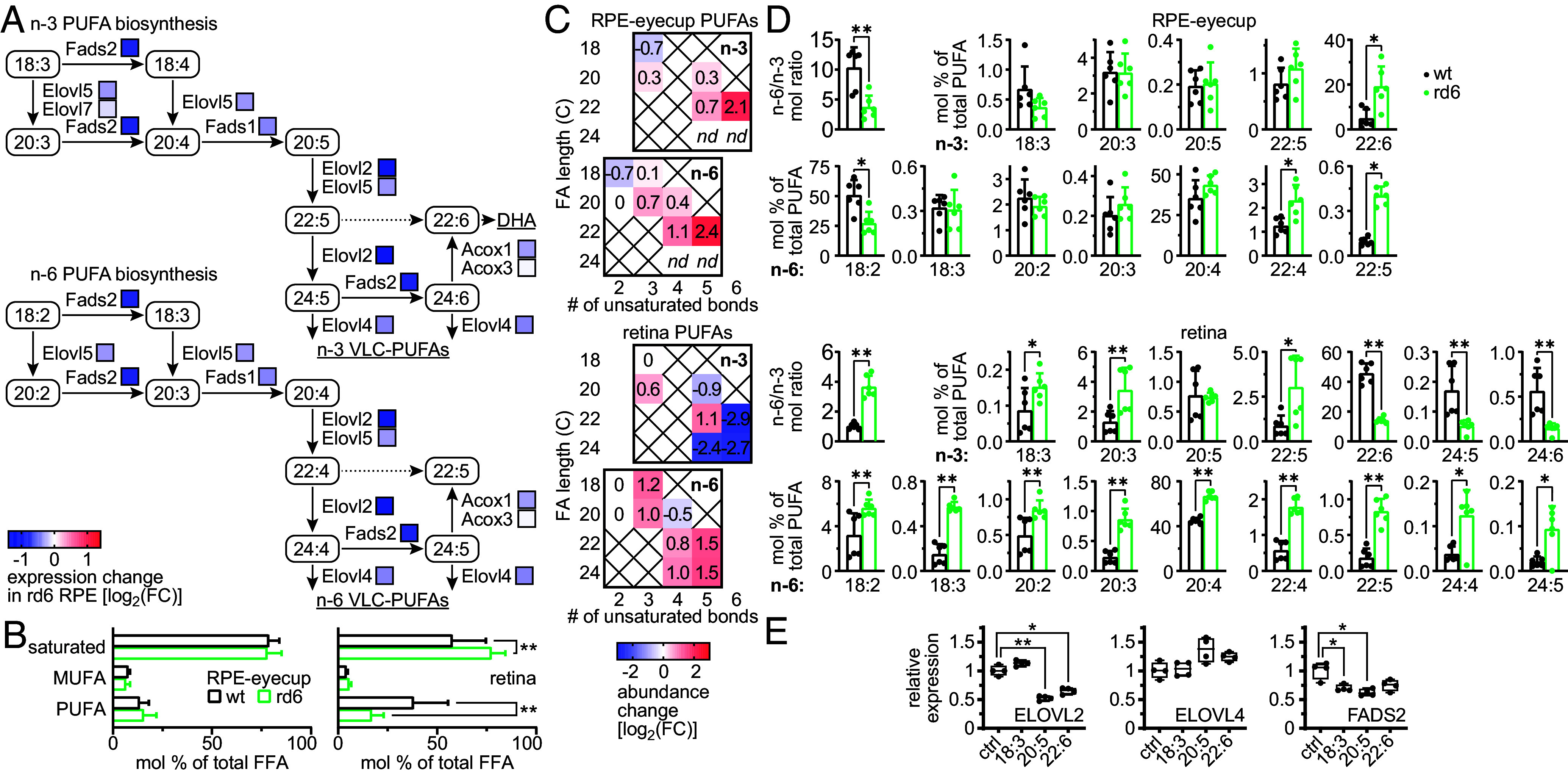
Analysis of PUFA metabolism in the MFRP-deficient eye. (*A*) Schematic representation of a PUFA biosynthetic pathway fragment, starting from α-linoleic acid (ALA) and LA, the major dietary sources of n-3 and n-6 PUFAs, respectively. Overlaid colored boxes depict the relative changes in expression of the respective genes, as observed for the rd6-mouse RPE cells in the transcriptomic analysis. (*B*) Lipidomic analysis of the FFA content in the RPE-lined eyecups and retinas of 1-mo rd6 and WT mice, divided into classes: saturated FA, MUFA, and PUFA. The bars represent mean ±SD of n = 6 independent replicates. Significant differences are indicated as follows: **P < 0.01, two-way ANOVA with the Holm–Šídák post hoc test. (*C*) Changes in the FFA composition in MFRP-deficient eyes. Heatmaps show fold-change in absolute abundance in samples from rd6 mice compared to WT. (*D*) Changes in the PUFA fractional composition in MFRP-deficient eyes. The first bar graph in each set of bar graphs shows the mol ratio (total free n-6/n-3 PUFA) from the analysis of RPE-eyecups (*Upper* set) or whole retina (*Lower* set). The remaining bar graphs in each set depict the percent composition of each PUFA molecular species in the total PUFA content. The bars represent the mean ± SD of n = 6 independent replicates. Significant differences are indicated as follows: **P < 0.01, *P < 0.05, Mann–Whitney test with correction for multiple comparisons. (*E*) qPCR-based analysis of ELOVL2, ELOVL4, and FADS2 gene-expression changes in a primary culture of bovine RPE, upon supplementation with an excess of the major substrate (ALA, 18:3) and products (EPA, 20:5 and DHA, 22:6) of the n-3 PUFA biosynthetic pathway, compared to untreated control (ctrl). The bars represent the mean ± SD of n = 4 independent replicates. Significant differences are indicated as follows: **P < 0.01, *P < 0.05, one-way ANOVA with the Holm–Šídák post hoc test.

Transcriptomic analysis revealed that the RPE cells in rd6 mice exhibit broad downregulation of the PUFA-biosynthesis pathway (Fig. 4*A*), including a significant, >2-fold decrease in the expression of ELOVL2 and FADS2. While both enzymes are involved in multiple stages of the endogenous synthesis of n-3 and n-6 long-chain PUFAs (up to 24 °C), they are exclusively responsible for the final steps of DHA biosynthesis: 22:5 to 24:5 fatty-acid chain elongation, and 24:5 to 24:6 Δ6 desaturation, respectively (Fig. 4*A*). DHA is the major PUFA in the retina, critical for photoreceptor development and function (32). Therefore, we further investigated the free-fatty-acid (FFA) composition of the eyes from 1-mo rd6 and WT mice, using an untargeted mass spectrometry (MS)-based approach. Analysis of samples derived from RPE-lined eyecups showed that loss of MFRP did not affect the overall ratios between saturated fatty acids, monounsaturated fatty acids (MUFAs), and PUFAs (Fig. 4*B*). However, we observed a ~2.8-fold decrease in the n-6/n-3 PUFA ratio, stemming largely from a significant accumulation of free DHA within the total FFA (Fig. 4*C*) and PUFA (Fig. 4*D*) fractions, and a corresponding reduction of the linoleic acid (LA, 18:2 n-6) content. We also observed significant accumulation of osbond acid (22:5 n-6), a low-abundance end product of the PUFA biosynthesis pathway, within the n-6 series. These observations suggest that DHA accumulation, rather than downregulation of its biosynthetic pathway, constitutes the primary defect caused by the MFRP deficiency.

In contrast, retinas from rd6 mice showed severe changes in overall FFA profiles compared to WT, including a significant decrease in total PUFA content, a compensatory increase in the abundance of saturated molecular species (Fig. 4*B*), and a >3-fold increase in n-6/n-3 ratio within the PUFA fraction (Fig. 4*D*). Underlying these events, we observed significant diminution of free DHA and its two n-3 precursors, tetracosapentaenoic (24:5) acid and tetracosahexaenoic (24:6) acid, within both the total FFA (Fig. 4*C*) and PUFA (Fig. 4*D*) fractions, and corresponding accumulation of the majority of n-6 PUFA species. While these results could indicate defects in PUFA supply from the RPE to the retina, such a significant loss of DHA (>7-fold compared to WT) at least partially reflects the progression of retinal degeneration in the rd6 mice, which lack about one-third of their DHA-rich photoreceptors at 1 mo of age (*SI Appendix*, Fig. S2).

For reference, we analyzed liver FFA profiles of the same animals. Here, we observed significant accumulation of osbond acid, and a similar, but not statistically significant, upward trend for several other 22C- and 24C-long n-6 PUFAs, correlating with a similar effect in the RPE (*SI Appendix*, Fig. S4). However, we recorded no significant changes in the n-3 series, suggesting that the DHA accumulation in the RPE constitutes a unique defect stemming specifically from MFRP loss.

To further evaluate whether a causative link exists between the accumulation of DHA and downregulation of the PUFA-biosynthesis pathway, we performed in vitro studies with primary cultures of bovine RPE. Supplementation of RPE cells with an excess of unesterified DHA or its early precursor eicosapentaenoic acid (EPA, 20:5) led to significant downregulation of both ELOVL2 and FADS2, but did not affect the expression of ELOVL4, the downstream enzyme in the pathway (Fig. 4*E*). In agreement, excess supply of the α-linolenic acid (ALA, 18:3 n-3), a primary dietary precursor of n-3 PUFAs, did not influence the expression levels of ELOVL2 or ELOVL4, enzymes not directly involved in ALA processing. This result further reinforced the idea that DHA accumulation in the RPE may be directly linked with the dysfunction of MFRP in this cellular layer, and lead to downregulation of the local PUFA-biosynthesis capacity.

### Loss of MFRP Leads to Severe Depletion of Di-DHA Phospholipids in the Retina.

**Fig. 5.**
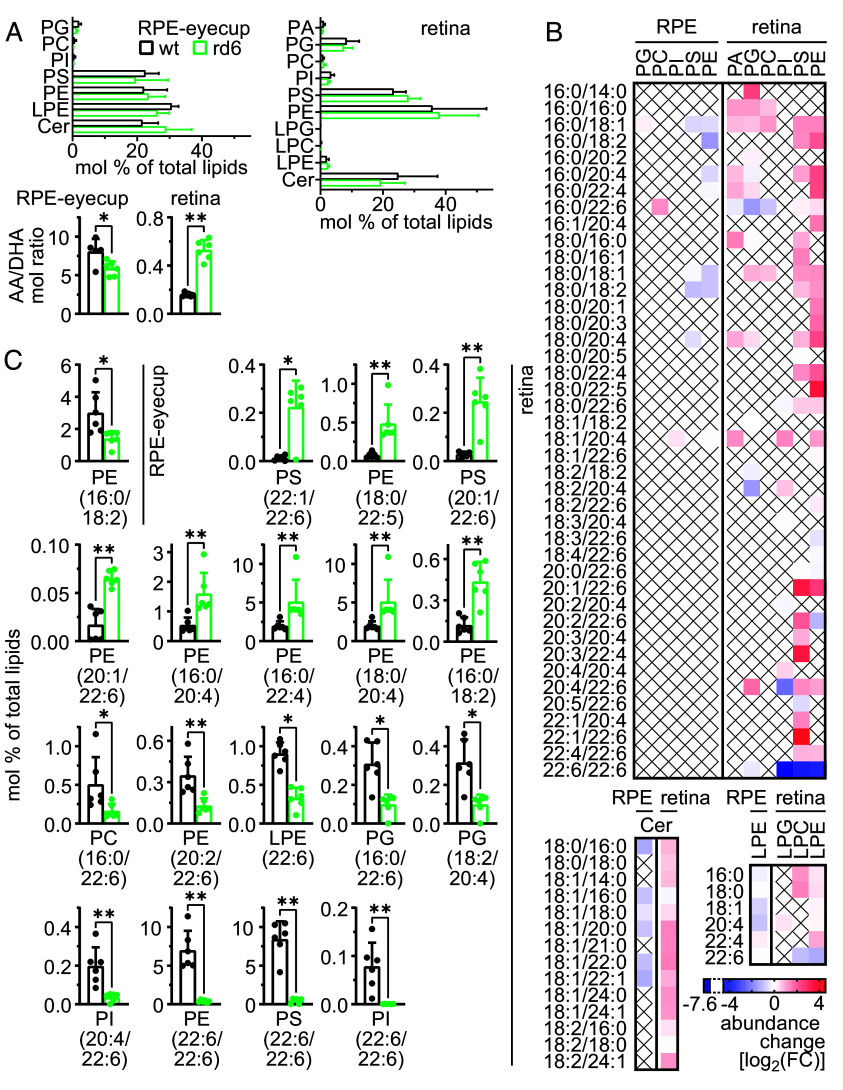
Changes in membrane-lipid content in the MFRP-deficient eye. (*A*) Lipidomic analysis of the phospholipid and sphingolipid content in the RPE-lined eyecups and retinas of 1-mo rd6 and WT mice. Upper graphs show relative content (mol %), and bottom graphs show AA/DHA molar ratios for the total quantified lipids. Each bar represents the mean ± SD of n = 6 independent replicates. Significant differences are indicated as follows: **P < 0.01, *P < 0.05, Mann–Whitney test with correction for multiple comparisons. (*B*) Overview of the changes in the lipid composition in MFRP-deficient eyes. Heatmaps show fold-change in absolute abundance in rd6 samples compared to WT. (*C*) Lipids exhibiting significant (≥2-fold, *P* < 0.05) change in abundance in the eyes from rd6 mice *vs*. WT. Graphs depict the percent composition of each molecular species of lipid in the total quantified lipid content. Each bar represents the mean ± SD of n = 6 independent replicates. Significant differences are indicated as follows: **P < 0.01, *P < 0.05, Mann–Whitney test with correction for multiple comparisons.

Based on the findings related to FFA metabolism we extended our analysis to full lipidomic profiling of the eyes from 1-mo rd6 and WT animals. Again, using the untargeted MS-based approach, we were able to reliably quantify 26 and 107 most-abundant membrane-lipid species in the RPE-eyecups and isolated retinas, respectively. The quantified lipids included several classes of phospholipids: phosphatidic acids (PA), phosphatidylserines (PS), phosphatidylinositols (PI), phosphatidylethanolamines (PE), phosphatidylcholines (PC), phosphatidylglycerols (PG), and lyso-forms of the latter three species (LPE, LPC, LPG, respectively); and a single class of sphingolipids: ceramides (Cer). In the RPE samples, we observed only minor changes associated with the MFRP loss. Lipid-class composition was not significantly altered between rd6 and WT samples; however, we observed a significant decrease in the AA/DHA ratio within the quantified lipids, corresponding to the changes observed at the FFA level (Fig. 5*A*). Focusing on individual lipids, the only significant change observed in the RPE was a 3.1-fold decrease of a single molecular species: PE(16:0/18:2) (Fig. 5 *B* and *C*).

In contrast, we observed profound lipidomic changes in the isolated retinas from the rd6 mice, compared to WT. These changes, evident at the level of single lipid species, did not affect the overall lipid-class composition, but resulted in a 3.3-fold increase in the AA/DHA molar ratio derived from all quantified lipids (Fig. 5*A*). Three lipids exhibiting the biggest decrease in the retinal samples were the di-DHA forms of three phospholipids: PI, PS, and PE (Fig. 5*B*). Their change in abundance, approx. 195-, 23-, and 18-fold decreases, respectively, occurred at a much greater magnitude than what could be explained exclusively by the 1/3rd diminution in the photoreceptor count, as is the case in retinas from 1-mo rd6 mice. Both PS(22:6/22:6) and PE(22:6/22:6) are also some of the most abundant species in WT retinas, each accounting for ~7 to 8% of the total lipid content (Fig. 5*C*). Thus, this observation suggests that MFRP in the RPE may specifically affect the retinal supply of di-DHA phosphoglycerides, which are considered to play an important role in phototransduction (33). Other lipids exhibiting significant (≥2-fold, *P* < 0.05) decreases included mostly mono-DHA species, such as PI(20:4/22:6), PG(16:0/22:6), LPE(22:6), PE(20:2/22:6), LPC(22:6), and PC(16:0/22:6). Prominent accumulation in the retina (>10-fold) was observed for three phospholipids, all incorporating either a single or no n-3 PUFA chain: PS(22:1/22:6), PE(18:0/22:5), and PS(20:3/22:4) (Fig. 5 *B* and *C*). No significant alterations were observed for the abundance of sphingolipids (ceramides) in RPE-eyecup and retinal samples.

### MFRP Selectively Binds Phosphatidylserine Lipid Species but Is not Involved in Photoreceptor Outer Segment Phagocytosis.

**Fig. 6.**
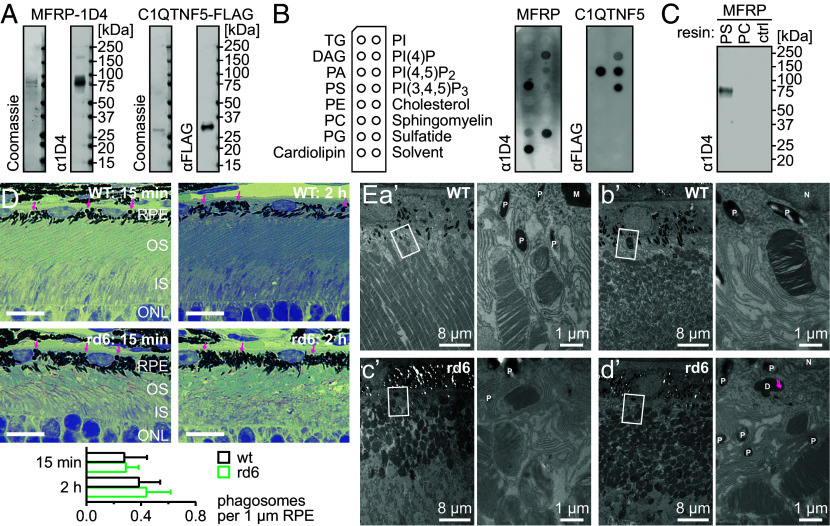
MFRP specifically binds PS and other lipids, but does not influence photoreceptor OS phagocytosis. (*A*) Reducing-SDS-PAGE analysis of purified recombinant human MFRP and C1QTNF5, harboring C-terminal 1D4 and FLAG epitopes, respectively. These tagged proteins were used in the lipid-binding assays shown in *B*. Protein identities and purity were verified by anti-1D4 or anti-FLAG immunoblotting, and Coomassie blue staining. (*B*) Binding of purified recombinant MFRP and C1QTNF5 to membrane lipids using a dot blot assay. Proteins were visualized by anti-1D4 or anti-FLAG immunodetection. (*C*) Affinity precipitation of recombinant MFRP on magnetic beads opsonized with PS, PC, or with no lipid (ctrl). Proteins bound to the resins were analyzed by immunoblotting. This experiment confirmed the specificity of the MFRP interaction with PS. (*D*) Light microscopy images of RPE–photoreceptor interfaces in 1-mo WT mice (*Top* row) and rd6 mice (*Bottom* row) from retinal sections stained with toluidine blue. Eyes for sectioning were sourced 15 min and 2 h after light onset. Phagosomes can be observed in the RPE layer (examples are indicated by pink arrows). Shortening of the OS structures in the rd6 animals is also detectable in these images. The bottom graph shows quantification of phagosome structures normalized to the length of the RPE layer within the section. Each bar represents the mean ± SD of n = 3 independent replicates. (Scale bar, 10 µm.) (*E*) TEM images of RPE–photoreceptor interfaces in 1-mo WT (*Top* row) and rd6 (*Bottom* row) mice. (*a*′) Photoreceptor OS and adjacent RPE cells in a vertically sectioned retina of a WT mouse (*Left* panel). The tip of the OS at the center of the image is phagocytosed and surrounded by microvilli of the RPE cell (rectangle). A high-magnification image of this region is shown (*Right* panel). Inside the RPE cell, there are electron-dense membranous structures (M), pigment granules (P), and endoplasmic reticulum. The membranous feature (M) likely corresponds to phagocytosed discs undergoing degradation. (*b*′) Photoreceptor OS and adjacent RPE cells in a tilted ultrathin retinal section of a WT mouse (*Left* panel). A small piece of OS is engulfed by the RPE cell (rectangle). A high-magnification image of this region is shown (*Right* panel). The endoplasmic reticulum, nucleus (N), and pigmented granules (P) of the RPE cell are observed around the engulfed photoreceptor outer segment. (*c*′) Photoreceptor OS and adjacent RPE cells in a tilted ultrathin retinal section from an rd6 mouse (*Left* panel). A piece of an OS was wrapped with microvilli of an RPE cell (rectangle). A high-magnification image of this region is shown (*Right* panel). (*d*′) Photoreceptor OS and adjacent RPE cells in a tilted ultrathin retinal section from an rd6 mouse (*Left* panel). A piece of photoreceptor outer segment is surrounded by RPE cell microvilli (rectangle region). A high-magnification image of the region is shown (*Right* panel). Inside the RPE cell, there is an electron-dense deposit (D) showing fine disc-membrane-like features (pink arrows). The endoplasmic reticulum, nucleus (N), and pigmented granules (P) of the RPE cell are observed around the deposit (D). In tilted sections (*b*′–*d*′), the OS and IS structures are observed as ovals due to the cutting angle.

The significant changes in lipid homeostasis upon MFRP loss of function evident from lipidomic profiling prompted us to study the lipid-binding properties of MFRP using recombinant protein produced in mammalian cells. As it has been shown previously that adiponectin-superfamily proteins, including C1QTNF5, a known MFRP ligand, bind selectively to certain phospho- and sphingolipids (34), we included C1QTNF5 as a positive control in our assay. Both the MFRP and C1QTNF5 proteins were obtained at >95% homogeneity, using an immunoaffinity purification approach (Fig. 6*A*). When assayed for membrane–lipid binding using dot blots, MFRP exhibited the highest affinity for PS, cardiolipin, and sulfatide. In contrast, C1QTNF5 showed almost no overlap in its lipid affinity, binding preferentially to PA and three phosphatidylinositol phosphates (PIP): PI(4)P, PI(4,5)P_2_, and PI(3,4,5)P_3_, suggesting specificity of the interactions observed for MFRP (Fig. 6*B*). Notably, both MFRP and C1QTNF5 displayed affinity for PI(4)P. Since PS plays a prominent role in the RPE–retina interface, being involved in initiation of the photoreceptor-OS-phagocytosis process, we further validated the MFRP interaction with PS. For this purpose, we prepared beads opsonized with either PS or PC and used them to precipitate recombinant MFRP. In agreement with previous observations, only PS-opsonized beads showed MFRP binding, confirming the specificity of this interaction (Fig. 6*C*).

Considering the inconsistent interpretations of the impact of MFRP loss on the phagocytosis process (13, 14), we further investigated the status of photoreceptor-OS phagocytosis in rd6 animals. Retinal sections from 1-mo mice prepared 15 min and 2 h after light onset, within the peak of phagocytic activity in the mouse eye (35), showed an abundance of phagosomes in both WT and rd6 RPE cells, stained with toluidine blue (Fig. 6*D*). We attempted to quantify the phagosomes based on their morphological characteristics, and observed no significant difference associated with MFRP deficiency at both time points tested (Fig. 6*D*). To confirm the occurrence of phagocytosis in the eyes of rd6 mice, we subjected retinal sections from 1-mo animals to transmission-electron-microscopy (TEM) imaging (Fig. 6*E*). Accordingly, we observed the presence of phagosomes containing membranous features reminiscent of phagocytosed discs undergoing degradation in both WT and rd6 RPE cells (Fig. 6 *E*, *a*′ and *d*′). Further supporting the evidence for active engulfment and uptake of OS tips, packets of well-aligned discs were surrounded and wrapped by apical membrane extensions of RPE cells in both mouse lines (Fig. 6 *E*, *b*′ and *c*′). Other lysosome-associated organelles, pigment granules, were similarly distributed in the cytoplasm of RPE in rd6 and WT mice. Despite previous reports that MFRP deficiency results in altered quantity and morphology of the RPE apical microvilli (13, 14), our morphological analysis of MFRP-deficient RPE cells showed no differences in the thickness of individual microvilli between WT and rd6 mice (Fig. 6*E*).

### MFRP Is Associated with ADIPOR1 and KCNJ13 in RPE Membranes.

**Fig. 7.**
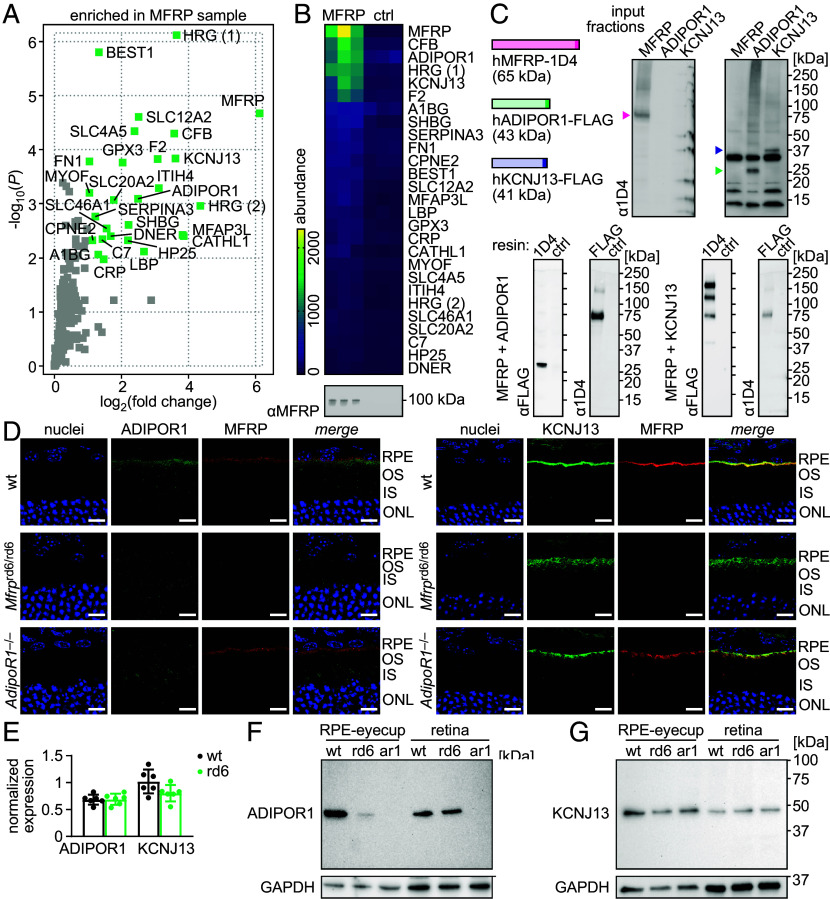
Analysis of the MFRP interactome. (*A* and *B*) Quantitative MS analysis of proteins interacting with MFRP in native bovine-RPE membranes. Proteins significantly (≥2-fold, *P* < 0.05) enriched in samples combined with anti-MFRP antibody compared to those combined with normal IgG controls (ctrl) are labeled green in the volcano plot (*A*); the heatmap (*B*) depicts their relative abundance in the analyzed samples. Successful immunoprecipitation of MFRP was confirmed by anti-MFRP immunoblotting. (*C*) Coimmunoprecipitation of recombinant human MFRP-1D4 with either ADIPOR1-FLAG or KCNJ13-FLAG. Input fractions and proteins bound to anti-FLAG, anti-1D4, or normal IgG (ctrl) resins, were analyzed by immunoblotting, which confirmed both pairwise interactions, MFRP–ADIPOR1 and MFRP–KCNJ13. (*D*) IHC images of eye cryosections from WT, *Mfrp*^rd6/rd6^ (rd6), and *AdipoR1*^–/–^ (ar1) animals on the albino (*Tyr^c-2J/c-2J^*) background, stained to visualize MFRP, ADIPOR1, and nuclei; or MFRP, KCNJ13, and nuclei. (Scale bar, 10 µm.) (*E*) Relative expression levels of ADIPOR1 and KCNJ13, normalized to ACTB, in the RPE samples from WT and rd6 mice. Each bar represents the mean ± SD of n = 6 independent replicates (RNA-seq analysis). Significant differences were not identified in unpaired *t* tests. (*F* and *G*) ADIPOR1 (*F*) and KCNJ13 (*G*) levels in protein extracts from preparations of RPE-eyecups and retinas from WT, *Mfrp*^rd6/rd6^ (rd6), and *AdipoR1*^–/–^ (ar1) animals. GAPDH served as a loading control.

It has been reported previously that MFRP deficiency in the RPE leads to the loss of ADIPOR1 from the apical membrane in this cellular layer (19). While this observation could be explained by a direct interaction between MFRP and ADIPOR1, information on the MFRP-protein interactome is limited to the validation of C1QTNF5 binding to MFRP in vitro (10, 16, 17). To identify potential MFRP-interacting partners in an unbiased manner, we performed affinity-purification-MS in detergent-free native bovine RPE membranes solubilized with styrene maleic anhydride (SMA) copolymers, which preserve native membrane-protein assemblies in lipid nano-disks. Comparison of proteins copurified on anti-MFRP resins *vs*. normal-IgG control resins showed significant (≥2-fold, *P* < 0.05) enrichment of 26 proteins in addition to MFRP (Fig. 7*A*). Among those of highest abundance (≥50% of MFRP level), we identified two other membrane proteins: ADIPOR1 and inward rectifier potassium channel 13 (KCNJ13) (Fig. 7*B*). Both proteins are expressed in the RPE, and their loss affects retinal health (36, 37). To verify both interactions, we performed coimmunoprecipitation with detergent-solubilized recombinant proteins containing FLAG (ADIPOR1, KCNJ13) or 1D4 (MFRP) epitopes, produced in the mammalian system. In agreement with the native-tissue data, both MFRP–ADIPOR1 and MFRP–KCNJ13 complexes were specifically pulled down with either anti-FLAG or anti-1D4 resins (Fig. 7*C*).

### MFRP Determines the Subcellular Localization of ADIPOR1 and KCNJ13 in Mouse RPE.

To further assess the biological relevance of MFRP interactions with its transmembrane-binding partners, we investigated their subcellular localization in retinas from WT and rd6 mice. In agreement with previous reports (19), ADIPOR1 was present in the RPE apical membranes of WT animals, but absent from this location in the rd6 strain. Notably, retinal distribution of MFRP appeared unaffected in ADIPOR1-deficient animals, suggesting that it is MFRP that specifically determines the subcellular localization of ADIPOR1 in the RPE cells (Fig. 7*D*). Analysis of the previously obtained RNA-seq data showed comparable levels of ADIPOR1 transcripts in the RPE samples from WT and rd6 mice (Fig. 7*E*). However, the protein level of ADIPOR1 was significantly decreased in the rd6 sample compared to the WT sample (Fig. 7*F*), indicating that the MFRP–ADIPOR1 interaction affects posttranscriptional processes specific for ADIPOR1, and possibly its trafficking to the RPE apical membrane. In contrast, immuno-staining for KCNJ13 revealed the presence of the potassium channel in the RPE–retina interface in all three conditions: WT, ADIPOR1-, and MFRP-deficiency. In both WT and ADIPOR1-knock-out animals, we observed almost complete colocalization of KCNJ13 and MFRP constrained to the RPE apical membrane, indicating that loss of ADIPOR1 does not influence the MFRP–KCNJ13 interaction. In contrast, MFRP loss resulted in diffuse distribution of KCNJ13, not limited to the apical membranes but including also the microvilli structures (Fig. 7*D*). In agreement, we did not observe differences in KCNJ13 transcript and protein abundance in RPE samples from WT and rd6 mice (Fig. 7 *E* and *H*), suggesting that the role of the MFRP–KCNJ13 interaction is limited to defining the fine subcellular localization of the latter, particularly in preventing its diffusion to the RPE apical processes.

### Recovery of MFRP Expression Restores Subcellular Distribution of ADIPOR1 and KCNJ13 in Mouse RPE.

**Fig. 8.**
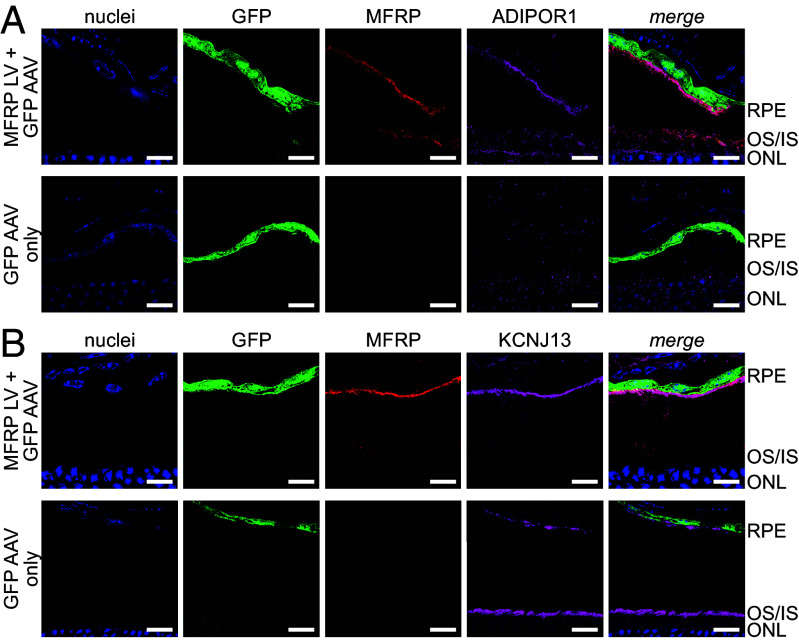
Recovery of MFRP expression in rd6 animals restores the correct subcellular localization of its binding partners. (*A* and *B*) IHC images of eye cryosections from *Mfrp*^rd6/rd6^ animals on the albino (*Tyr^c-2J/c-2J^*) background that were injected subretinally with lentivirus encoding the WT variant of MFRP, along with a GFP-expressing AAV. Samples were stained to visualize MFRP, ADIPOR1, and nuclei (*A*); and MFRP, KCNJ13, and nuclei (*B*). Restoration of MFRP expression restores ADIPOR1 in the RPE and corrects the subcellular localization of KCNJ13 within the RPE apical membrane. (Scale bar, 10 µm.)

Novel gene therapy approaches hold promise for curing a major portion of inherited diseases, including in the eye (38). Several gene-augmentation (4–8) and gene-editing (9) trials have shown success in preserving morphology of the retina and improving its visual function in mouse models of MFRP deficiency. Accordingly, we further investigated whether recovery of MFRP expression in the RPE would restore the molecular hallmarks of its loss; i.e., correct subcellular distribution of its binding partners. For this purpose, we performed subretinal injections into 1-mo rd6 mice to administer a lentivirus encoding the WT variant of MFRP. A GFP-expressing adeno-associated virus (AAV) was coinjected to enable the assessment of transfection efficiency. At 3 wk postinjection, we performed fundus imaging with scanning laser ophthalmoscopy (SLO), and eyes exhibiting GFP expression covering >80% of the RPE were further sectioned and analyzed by immunohistochemistry. As expected, eyes from rd6 mice supplied with the WT copy of MFRP showed robust restoration of its expression and its subcellular localization within the apical membrane of RPE cells. Additionally, we observed that the distribution of both ADIPOR1 and KCNJ13 was restored to the WT state (Fig. 8), confirming efficacy of the gene-therapy at the molecular level.

To gain further insights into the function of MFRP, we designed several additional lentiviruses encoding MFRP variants harboring different single-domain deletions (*SI Appendix*, Fig. S5*A*). We successfully validated production of the following mutant MFRP variants in vitro: ΔCUB-1, ΔCUB-2, ΔLDLa-2, and ΔFz (*SI Appendix*, Fig. S5*B*). Next, we performed rescue experiments with WT MFRP on 1-mo rd6 animals in a fashion similar to the previously described gene-therapy trial. Notably, all of the MFRP variants were able to restore the presence and correct subcellular localization of ADIPOR1 within the apical RPE membrane, suggesting that the amino acid residues determining both MFRP subcellular trafficking and the MFRP–ADIPOR1 interaction are located outside of the altered MFRP domains (*SI Appendix*, Fig. S5*C*). This observation also suggests that MFRP has modular architecture, whereby removal of individual structural domains does not alter the overall protein structure in a way sufficient to trigger its instant degradation.

## Discussion

MFRP expression occurs relatively late in human ocular development, around the second trimester of gestation, and affects prenatal and postnatal axial growth of the eye, including the process of emmetropization (11). Mutations in the MFRP gene cause nanophthalmos, a developmental defect manifested by abnormally short axial eye length, in both humans and mice (6). This defect is accompanied by retinal dysfunction, affecting rod photoreceptors primarily. However, while evident early in life in the animal models, its presence and severity among patients varies and may depend on the age of the person as well as the underlying genetic variant (39). One of the possible explanations for this variation could be that MFRP plays more than one role in eye biology, depending on factors such as developmental stage, spatial distribution between different cell types (RPE, ciliary body, iris), or the modular architecture of the protein with its several different functional domains.

On the molecular level, MFRP deficiency leads to a plethora of changes in the RPE and adjacent retina. The RPE cells display evidence of widespread activation of humoral and complement immune responses and decline in some of their major functions, such as slow kinetics of visual chromophore regeneration, and likely decreased DHA uptake and transport to the retina due to downregulation of MFSD2A and ACSL6 (40, 41). Indeed, lipid biosynthesis in the RPE and overall lipid homeostasis are severely affected by loss of MFRP. In contrast, another function of the RPE critical for retinal health, daily phagocytosis of photoreceptor OS, seems unaffected in the absence of MFRP. This dichotomy indicates that loss of MFRP has only compartmental impact on overall RPE biology. Notably, all of the observed changes are composed of primary effects of loss of MFRP function, and secondary effects, likely compensatory, influenced by the degenerating retina. Moreover, as all of the observed lipid-related changes in the MFRP-deficient RPE seem unlikely to lead to DHA accumulation, we conclude that DHA accumulation likely constitutes one of the primary effects of MFRP loss. Accordingly, we confirmed in vitro that at least one aspect of the MFRP-deficient phenotype in the RPE, downregulation of the FFA biosynthesis pathway, could be caused by the other aspect, DHA accumulation in the cell.

In this study, we established several molecular properties of MFRP, gaining some insights into what the MFRP functions could be. We showed that MFRP undergoes extensive glycosylation, and likely possesses a juxtamembranous OLS domain. Such a stretch of O-glycosylated amino acids protruding from the cell surface has been identified previously in the extracellular portions of other membrane proteins, including low-density and very low-density lipoprotein receptors (LDLR and VLDLR, respectively), and decay-accelerating factor (DAF; CD55) (42). Furthermore, OLS domains have been shown to serve as rigid, protease-resistant spacers at the cell surface (26). In this regard, it is pertinent that the *MFRP*-gene mutation carried by rd6 mice leads to an in-frame deletion of 58 amino acids located between the transmembrane and first CUB domains (1), which includes the entire OLS region, highlighting its importance for MFRP function.

We also showed that MFRP has at least two membrane-binding partners, ADIPOR1 and KCNJ13; however, separate disruption of these interactions has different molecular consequences, suggesting different functions. Disappearance of ADIPOR1 protein from the RPE upon loss of MFRP has been observed previously (19). The fact that ADIPOR1-transcript levels are not affected in rd6 animals implies that the MFRP–ADIPOR1 interaction determines the posttranslational fate of ADIPOR1. The lipidomic hallmarks of MFRP deficiency, accumulation of DHA in the RPE cells and severe diminution of di-DHA phospholipids in the retina, are recapitulated in global ADIPOR1-knock-out animals (20, 43, 44). In addition, there is extensive phenotypic similarity between the two knock-out mouse models, including the speed of photoreceptor death progression, the presence of macrophages, and spot-wise accumulation of autofluorescent material across the fundus. Overall, these parallel effects suggest that loss of MFRP leads to a complex phenotype, a part of which being RPE-specific ADIPOR1 deficiency. Whether loss of this interaction underlies the lipid-associated phenotype in rd6 animals remains to be tested.

In contrast to ADIPOR1, protein levels of KCNJ13 in the RPE remain unaffected by MFRP loss. The KCNJ13 channel is present in the RPE–retinal interface; however, instead of being enriched in the RPE apical membrane as observed in WT animals, its subcellular localization in the rd6 mice is shifted toward the apical microvilli. This observation indicates that MFRP does not influence the posttranslational processes involving KCNJ13, including its targeting to the apical side of RPE cells; rather, MFRP limits diffusion of KCNJ13 to the microvilli membranes. The physiological consequence of such a distributional change remains unclear; however, one might speculate that the normal presence of the channel in the thin and extended microvilli could be integral to the morphology of the microvilli and impact the kinetics of potassium efflux into the extracellular environment. Accordingly, MFRP-deficient animals have been reported to exhibit altered quantity and morphology of the RPE apical microvilli (13, 14); however, we did not observe such changes in our own TEM analysis. In addition, potassium concentration changes are a major factor regulating ocular growth rates and hence the emmetropization process (45–47). Since KCNJ13 mutations have not been linked with nanophthalmia, rather leading to more severe retinal disorders (Leber congenital amaurosis and snowflake vitreoretinal degeneration), the potential role of the MFRP–KCNJ13 interaction in ocular development and homeostasis remains unresolved and also requires further investigation.

A final observation related directly to the molecular properties of MFRP is that it exhibits preferential binding to lipids of several classes, including PS, a major component of plasma membranes; and several minor components, PI(4)P, cardiolipin, and sulfatide. The localization of MFRP to the apical RPE membrane and its binding to PS prompted us to reevaluate its influence on OS phagocytosis; however, as noted above, MFRP appears not to be involved in this process. However, the affinity of MFRP to certain lipids may explain its potential role in posttranslational processing and trafficking to the apical side of RPE cells. Indeed, PI(4)P is a known regulator of export of proteins from the Golgi apparatus and their subsequent secretion (48). Since MFRP undergoes extensive glycosylation, likely in the Golgi compartment, but lacks any apparent signaling sequence for subcellular localization, it is plausible that binding to PI(4)P and other lipids affects its intracellular trafficking. In line with this theory, subcellular localization of MFRP and ADIPOR1 is not affected by individual deletions of its CUB, second LDLa, or Fz domains. It is therefore possible that MFRP affects the composition of the RPE apical membrane by directing its interacting proteins and lipids as a cargo through its PI(4)P-binding-driven subcellular-trafficking pathway. Overall, MFRP appears to play the role of a molecular hub that organizes the apical membrane of RPE cells. It specifically binds certain proteins, including C1QTNF5, ADIPOR1, and KCNJ13, and affects subcellular localization of the two latter. MFRP also binds specific lipids, but the biological significance of those interactions remains unknown and requires further investigation.

## Materials and Methods

### Experimental Animals.

All animal procedures followed the ARVO Statement for the Use of Animals in Ophthalmic and Vision Research and the NIH Guide for the Care and Use of Laboratory Animals; and they were approved by the Institutional Animal Care and Use Committee of UC Irvine (IACUC protocol AUP-24-073), and Indiana University. The following previously described mouse strains were used in this study: C57BL/6 J, B6(Cg)-*Tyr^c-2J^/J*, *Mfrp^rd6^/J* (Jackson Laboratory), and B6.129P2-*Adipor1^tm1Dgen^*/Mmnc (Mutant Mouse Resource and Research Center). For some experiments *Mfrp^rd6^/J* and B6.129P2-*Adipor1^tm1Dgen^*/Mmnc backcrossed into the B6(Cg)-*Tyr^c-2J^/J* (albino) background were used. Animals were housed under 12-h/12-h light/dark cycles and fed a standard soy protein-free diet (Teklad 2020X, Envigo) ad libitum. Unless otherwise noted, both male and female mice were used for all in vivo and in vitro experiments. All animals were drug- and test-naïve.

### Lentivirus Production.

The cDNAs of mouse MFRP (full length and deletion variants) with C-terminal 3× FLAG epitope in the pTwist Lenti SFFV vector were obtained from Twist Biosciences. Lentivirus particles were produced as previously described (71), using HEK293T/17 cells cultured in DMEM with 10% FBS.The day before transfection, 10^7^ cells were plated per 150-cm dish. On the day of transfection, each dish was supplemented with a mixture of 120 µg PEI max (Polysciences) with 8 µg of pCMV VSV-G (Addgene), 16 µg of psPAX2 (Addgene), and 16 µg of transfer genomes (pTwist Lenti SFFV) in OptiMEM (Thermo Fisher Scientific). 48 h after transfection, medium was collected, clarified by centrifugation at 500*g* for 5 min at RT, and filtered through a 0.45 µm filter. Lentivirus particles were then pelleted by ultracentrifugation (100,000*g*?) with a 20% sucrose in PBS (w/v) cushion, and resuspended at a concentration factor of 1,000-fold in 10% sucrose in PBS (w/v) before distributing aliquots into LoBind tubes (Corning), and slow freezing in a Mr. Frosty (Thermo Fisher Scientific) isopropanol freezing container. The titer of lentivirus particles was determined, using the Lenti-X GoStix test (Takara Bio); they were stored at −80 °C and thawed immediately before use.

### Quantification and Statistical Analysis.

Experiments were replicated at least three times using distinct biological samples, with sample sizes (n) and what they represent noted in figure legends. Quantitative data are presented as mean ± SD or SEM, as indicated. Statistical analysis was performed using Prism 10 software (GraphPad). *P* values less than 0.05 were considered statistically significant. Data were tested for normal distribution using the Shapiro–Wilk test followed by evaluation using an appropriate test. The Mann–Whitney test or unpaired t test was performed for comparisons between two groups of samples. Experiments involving ≥3 groups of samples with normally distributed data were analyzed using one-way or two-way ANOVA. Post hoc tests to account for multiple comparisons utilized the Holm–Šídák approach (with ANOVA) or the Benjamini, Krieger, and Yekutieli approach (with Mann–Whitney tests). Details of the statistical analyses applied for particular comparisons are indicated in the figure legends.

## Supplementary Material

Appendix 01 (PDF)

## Data Availability

MS proteomics data, and sequencing data have been deposited in Mass Spectrometry Interactive Virtual Environment (MassIVE) repository, and Gene Expression Omnibus (GEO) database (MSV000096610 and GSE283089, respectively) (49, 50).
