# Supplementary material for: MFRP is a molecular hub that organizes the apical membrane of RPE cells by engaging in interactions with specific proteins and lipids
Source: Proc Natl Acad Sci U S A. 2025 Apr 18;122(16):e2425523122. doi: 10.1073/pnas.2425523122 (PMC12036977; doi:10.1073/pnas.2425523122)
Supplement: Supplementary file 1 — Appendix 01 (PDF) [file pnas.2425523122.sapp.pdf]

PNAS

Supporting Information for

MFRP is a molecular hub that organizes the apical membrane of RPE cells by engaging in interactions with specific proteins and lipids.

Aleksander Tworak, Roman Smidak, Carolline Rodrigues Menezes, Samuel W Du, Susie Suh, Elliot H Choi, Sanae S Imanishi, Zhiqian Dong, Dominik Lewandowski, Kristen E Fong, Gabriela Grigorean, Antonio F. M. Pinto, Qianlan Xu, Dorota Skowronska-Krawczyk, Seth Blackshaw, Yoshikazu Imanishi, Krzysztof Palczewski

Corresponding author: Krzysztof Palczewski
Email: kpalczew@uci.edu

This PDF file includes:

Supporting text: Materials and methods
Figures S1 to S5
Tables S1 to S5
SI References

Materials and methods

***In silico* analysis.** For *in silico* analyses, human genome hg38 and mouse genome mm10 assemblies in the UCSC genome browser (1) were used. RNA-seq coverage data were retrieved from the Genotype-Tissue Expression (GTEx) Portal (2), annotation of DNA regulatory elements originates from the ENCODE project (3), and alternative splicing data were retrieved from ASCOT (4). Protein sequence annotation was retrieved from InterPro (5), and glycosylation site predictions were performed using NetNglyc 1.0 server (6) for N-glycans and NetOglyc 4.0 server (7) for O-glycans. Human single-cell expression data were analyzed and visualized in Single Cell Portal (8), using the SCP2310 study (9). The molecular-structure model of human MFRP was obtained from the AlphaFold Protein Structure Database (10), and visualized using ChimeraX (11).

Immunohistochemistry. Human retinal tissue sections were obtained from a donor (age 83) with appropriate consent from the San Diego Eye Bank, following a protocol approved by the University of California, San Diego Human Research Protection Program. The donor had had no history of eye disease, diabetes, or any neurological diseases. For mouse specimens, enucleated mouse eyes were rinsed in PBS and incubated for 10 min at RT in freshly prepared fixing solution (PBS with 4% paraformaldehyde, Electron Microscopy Sciences). After dissection along the posterior margin of the limbus, lens and vitreous were removed. To prepare RPE flatmounts, four radial cuts were taken on each dissected eyecup, the retina was removed, the remaining eyecup was flattened on a glass slide, incubated in the fixing solution for 20 min at RT, washed three times with PBS, and immediately used for immunostaining. To prepare sagittal sections of the posterior eye segment, the dissected eyecup was further incubated in the fixing solution for 20 min at RT, rinsed in PBS and subjected to three brief washes in 5% (w/v) sucrose (MilliporeSigma) in PBS, two 30-min incubations each in the 10% (w/v) and 20% (w/v) sucrose-PBS solutions, and an overnight incubation at 4°C in the embedding medium (2:1 (v/v) mixture of 20% (w/v) sucrose solution in PBS and Tissue-Tek O.C.T. Compound; Sakura). Following the embedding and freezing on dry ice, the eyecup was cut into 12-µm-thick sagittal sections with a CM1850 cryostat-microtome (Leica Biosystems), placed on glass slides, and stored at -80°C until needed. For immunostaining of RPE flatmounts or defrosted and rehydrated retinal sections, the samples were incubated for 1 h at RT in blocking buffer (PBS with 3% (w/v) bovine serum albumin, MilliporeSigma; 3% normal donkey serum, MilliporeSigma; and 0.1% Triton-X100, MilliporeSigma). Samples were subsequently incubated overnight at 4°C in the blocking buffer with the appropriate primary antibodies, as listed in Table S4. After three washes with PBS

containing 0.1% Triton-X100 (PBS-T), the samples were incubated for 1 h at RT in blocking buffer with secondary antibodies (Table S4), followed by PBS with DAPI nuclear stain (Thermo Fisher Scientific) for 15 min at RT. Next, samples were washed three times with PBS-T and mounted with ProLong Glass Antifade medium (Thermo Fisher Scientific) for imaging. Fluorescence images were acquired using BZ-X800 (Keyence) and Elyra 7 (Zeiss) microscopes at low and high magnification, respectively.

***In vivo* retina imaging.** Following pupil dilation with 1% tropicamide (Akorn), mice were anesthetized with an IP injection of ketamine/xylazine solution (100/10 mg/kg). A Bioptigen *in vivo* spectral-domain OCT device (Leica Microsystems) was used to perform rectangular scans at a rate of 1200 A-scans/B-scan. For each eye, an average of five repeated B-scans centered on the optic nerve head (ONH) and acquired at 0 and 90 were used for analysis. Retinal ONL thickness was measured 500 μm away from the ONH in four retinal quadrants (superior, inferior, nasal, temporal), and further averaged to give an overall value per eye. SLO was performed using a Heidelberg Retinal Angiograph II (Franklin) in the autofluorescence mode, and acquired images were analyzed qualitatively.

***In vivo* ERG.** Mice, dark-adapted overnight, were anesthetized with an IP injection of a mixture of ketamine/xylazine solution (100/10 mg/kg), and their pupils were dilated with a drop of 1% tropicamide. The temperature of each mouse's body was maintained at 37°C with a heating pad. ERG responses were measured from both eyes by contact corneal electrodes held in place by a drop of Gonak solution, using the UTAS E-3000 system (LKC Technologies). For single-flash scotopic ERG recording, the duration of white-light flash stimuli (from 20 μsec to 1 msec) was adjusted to provide a range of illumination intensities from −3.7 to 2.3 log (cd·s/m2). For each intensity, 3 to 20 recordings were made at sufficient intervals between flash stimuli (from 3 to 90 sec) to allow recovery from any photobleaching effects. The photopic ERG recordings were performed after bleaching at 1.4 log (cd·s/m2) for 10 min. For photopic ERG, the cone response was measured at four different light intensities (−0.7 to 2.3 log(cd·s/m2)) in the presence of rod-desensitizing white-light background.

Histology. The enucleated mouse eyes were kept in Hartman's fixative (MilliporeSigma) for 24 h at RT, then transferred to 70% ethanol for further processing by the Visual Sciences Research Center Core facility at Case Western Reserve University. The eyes were embedded in paraffin and sagitally cut into 6-μm sections. Sections spanning the ONH were stained with hematoxylin and eosin and imaged by light microscopy with Olympus FSX100 and Keyence BZ-X800 instruments. Manual counting of photoreceptor nuclei per row, every 300 μm starting from the

edge of the ONH along both superior and inferior directions, was performed using QuPath software (12). Average values of ONL-nuclei counts for each animal group represent data obtained from 5 eyes.

RNA sequencing and data analysis. To isolate RPE from the enucleated mouse eyes, the anterior segment and the neural retina were removed from the eyecup, which was then incubated in RNAprotect cell reagent (Qiagen), following a previously established protocol (13). Total RNA was isolated from the combined RPE from both eyes, using a RNeasy Micro kit (Qiagen) according to the manufacturer's directions, then analyzed using the Bioanalyzer assay (Agilent) to ensure good quality (RNA integrity number, RIN ≥8). RNA samples were processed in the Transcriptomics and Deep Sequencing Core (Johns Hopkins University) for library preparation and sequencing. Briefly, total RNA (~100ng per sample) was used for library preparation by following the Illumina TruSeq Stranded Total RNA Library Prep Kit protocol. Libraries were then sequenced (paired-end 100 cycles) with the Illumina NextSeq 500 platform, yielding approximately 40 million raw reads per library. Sequence data were de-multiplexed using bcl2fastq2 software (Illumina), and their quality was assessed using FastQC (Babraham Bioinformatics). Reads were aligned to the reference mouse genome assembly (mm10) and quantified using the Rsubread software package with standard parameters (14). Data normalization and differential expression analyses were performed with DESeq2 (15), with a Benjamini–Hochberg adjusted *P*-value (*P*adj) <0.05 considered as statistically significant. To test the enrichment of biological signatures, the expression data were analyzed with Gene Set Enrichment Analysis (GSEA) (16), using two gene-set collections from the Molecular Signatures Database (MSigDB, v7): C2 curated gene sets, including Reactome (17) and Kyoto Encyclopedia of Genes and Genomes (KEGG) pathways (18); and C5 gene ontology (GO) (19) gene sets. GSEA was performed using 1000 permutations by gene set, with weighted enrichment statistic; and genes were ranked by a signal to noise metric. Gene sets with false discovery rate (*q*) <0.01 were considered significantly associated with the phenotype of interest. Raw data generated in this study were submitted to Gene Expression Omnibus (GEO) database (accession number GSE283089).

Retinoid profiling. Analyses were performed following a previously established protocol (20). Briefly, mice were either dark-adapted for 16 h, or subjected to photo-bleaching (5000 lux light for 10 min) followed by a specified regeneration period in the dark (2, 4, 6, 8, or 20 h). Further procedures were performed in the darkroom under dim red light. Enucleated eyes were flash frozen (2 per sample) and stored at -80°C for further use. For retinoid extraction, eyes were

homogenized in 1 mL of 10 mM sodium phosphate buffer, pH 8.0 with 50% methanol (v/v), and 100 mM hydroxylamine, and vigorously mixed with 2 mL of 5 M NaCl and 4 mL of methyl-tert-butyl-ether. The organic layer was dried *in vacuo* and reconstituted in 300 µL of heptane. The resulting suspension was centrifuged at 20,000*g*, and 100 µL of extract was injected for normal-phase HPLC analysis (Agilent Zorbax Rx-Sil 5 µm, 4.6 x 250 mm; Agilent Technologies) in a stepwise gradient of ethyl acetate in hexanes (0 – 17 min, 0.5%; 17.01-50 min, 10%) at a flow rate of 1.4 $mL \cdot min^{-1}$. Retinoids were detected by monitoring absorbance at 325 nm for retinyl esters and retinol, and at 360 nm for retinyloximes. Retinoids were quantified based on standard curves relating the chromatographic peak area to the molar amount of each retinoid standard.

Lipid profiling. Enucleated mouse eyes were dissected along the posterior margin of the limbus, and the lens and vitreous were removed; retinas and RPE-eyecups were separated, flash frozen (4 per sample), and stored at -80°C for further use. Lipidomic analysis was performed in the MS Core at the Salk Institute. Lipids were extracted using a modified version of the Bligh-Dyer method (21). Briefly, each tissue sample was homogenized with a probe sonicator in 1 mL of PBS, transferred to glass vials (VWR), mixed with 1 mL of methanol and 2 mL of chloroform, containing $^{13}C16$-palmitic acid internal standard; the mixture was shaken for 30 sec, vortexed for 15 sec, and centrifuged at 2400*g* for 6 min. The bottom organic layer was retrieved, dried under a gentle stream of nitrogen, and reconstituted in a 2:1 (v/v) mixture of chloroform and methanol. LC-MS analysis was performed on a Vanquish UHPLC system interfaced with a Q-Exactive quadrupole-orbitrap mass spectrometer with an electrospray ion source (Thermo Fisher Scientific). Lipids were separated using a Bio-Bond C4 column (5 µm, 4.6 x 50 mm; Dikma) and two solvents: 95:5 (v/v) mixture of water and methanol (solvent A), and 60:35:5 (v/v/v) mixture of isopropanol, methanol, and water (solvent B). MS data were acquired in negative ionization mode, where solvents A and B contained additionally 0.028% ammonium hydroxide. A solvent gradient was used as follows: 0-5 min 0.1 mL/min 0% B, 5-55 min 0.4 mL/min linear gradient 20-100% B, 55-63 min 0.5 mL/min 100% B, 63-70 min 0.5 mL/min 0% B. Spray voltage was 3.5 kV and 2.5 kV for positive and negative ionization modes, respectively. Sheath, auxiliary, and sweep gases were 53, 14, and 3 units, respectively. The capillary temperature was 275°C. Data were collected in full MS/dd-MS2 mode (top 5). Each full mass spectrum was acquired from 150–1500 m/z with a resolution of 70,000, AGC target of $1x10^6$, and a maximum injection time of 100 msec. MS2 was acquired with a resolution of 17,500, a fixed first mass of 50 m/z, AGC target of $1x10^5$, and a maximum injection time of 200 msec. Stepped normalized collision energies were 20, 30, and 40%. Lipid identification was performed with LipidSearch (Thermo Fisher Scientific) and verified

with Skyline (22); quantification involved integration of Skyline peak areas and normalization to the tissue weight and internal standard.

Bovine RPE isolation. Fresh bovine eyes, procured from a local abattoir within three hours post-mortem, were hemisected and the anterior portion, vitreous, and retina were removed following a previously established protocol (23). RPE cells were isolated by gentle brushing in ice-cold 20 mM HEPES pH 7.4, 0.25 M sucrose, 1 mM tris(2-carboxyethyl)phosphine. The suspension was passed through cotton gauze to remove larger pieces of tissue and centrifuged for 5 min at 1,000*g* at 4°C. Cells were stored at -80°C.

Primary bovine RPE culture. All procedures were performed in a cell culture-rated biosafety cabinet. Fresh bovine eyes, procured from a local abattoir within three hours post-mortem, were cleaned from accessory tissues encompassing the globes, immersed in 70% ethanol for 30 seconds, and rinsed with PBS. Eyes were hemisected and the anterior portion, vitreous, and retina were removed following a previously established protocol (23). The remaining eye cups were washed with PBS, filled with 0.25% Trypsin-EDTA solution (Thermo Fisher Scientific), and incubated for 30 min in 37°C, 5% CO_2. Next, RPE cells were dislodged from the inner surface of the eye cups *via* gentle pipetting, the cell suspension was mixed with fetal bovine serum (FBS, Genesee Scientific) at a 9:1 (v/v) ratio and centrifuged for 20 min at 500*g*. The pelleted cells were washed twice with PBS, resuspended in the culture medium (RtEGM Retinal Pigment Epithelial Cell Growth Medium BulletKit; Lonza), adjusted to a density of 10^5 cells per ml, dispensed into 10-cm tissue-culture dishes (Corning) at 1.5 x 10^6 cells per dish, and cultured under 5% CO_2 at 37°C. After 24 h, cells were washed twice with PBS and supplied with fresh culture medium. After a week, cells were sub-cultured at a 1:3 dilution with fresh medium and used for further experimental procedures.

Lipid supplementation assay. DHA, EPA, and ALA (Cayman Chemical Company) were each dissolved in 100% ethanol (MilliporeSigma) and mixed with fatty acid-free bovine serum albumin (FA-free BSA; MilliporeSigma) in PBS at a 10:1 molar ratio. These mixtures were incubated for 1 h at 37°C with delicate shaking, then mixed with fresh RPE culture medium at 250 µM final FFA concentration, and added to the RPE cell cultures. After 72 h in standard culture conditions FA-treated and untreated cells were washed with PBS, harvested by scraping off the dish surface, collected by centrifugation for 5 min at 200*g*, and immediately processed using RNeasy Micro total RNA isolation kit (Qiagen) with DNase treatment. For quantitative analysis of the mRNA levels of FADS2, ELOVL2, ELOVL4, and GAPDH, the cDNA transcripts were prepared from RNA with the iScript Reverse Transcription kit (Bio-Rad) and used for real-time PCR with iTaq Universal

SYBR Green Supermix (Bio-Rad), according to the manufacturer's protocol. The primers are listed in Table S5. A CFX384 Real-Time PCR Detection System (Bio-Rad) was used under the following conditions: 95°C for 30 s, followed by 40 cycles 95°C for 5 s, and 60°C for 30 s. Gene expression was normalized to the GAPDH-mRNA levels in each sample, and ΔCT and relative expression were evaluated as $2^{-\Delta CT}$.

Recombinant protein production. Full-length cDNAs of human MFRP, ADIPOR1, C1QTNF5, and KCNJ13 were obtained from Genscript, each with a C-terminal FLAG (DYKDDDDK) epitope in the pcDNA3.1 vector for mammalian cell expression. In the MFRP coding sequence the C-terminal FLAG was replaced with a 8-His and 1D4 (TETSQVAPA) epitope, using standard PCR cloning methods. Expression was carried out in Expi293F (GnTI-) cells (Thermo Fisher Scientific), grown in Expi293-expression medium (Thermo Fisher Scientific), following the manufacturer's protocol. Cells were transfected using ExpiFectamine 293 (Thermo Fisher Scientific). For membrane proteins, cells were collected after 48-72 h by centrifugation for 5 min at 300*g*, and stored at -80°C. For C1QTNF5, culture-conditioned medium was collected on day 5 post-transfection, filtered, and stored at 4°C until isolation.

Membrane isolation. All procedures were performed at 4°C, and all solutions contained Complete-Ultra protease-inhibitor cocktail (Roche). Cell pellets were suspended in 20 mM Tris pH 7.4, incubated 30 min with rotation, and centrifuged for 10 min at 3,200*g*. Pellets were resuspended in 20 mM HEPES pH 7.4, 250 mM sucrose. Cells were lysed either by two passes through a French pressure cell (Expi293), or ten passes through a 26G needle (bovine RPE). Sucrose concentration in the lysates was adjusted to 1.35 M using a solution of 2.5 M sucrose in 20 mM HEPES, pH 7.4. Lysates were then transferred to open-top 13.2-ml tubes (Beckman Coulter), overlaid with 20 mM HEPES pH 7.4 and 150 mM NaCl, and centrifuged for 30 min at 230,000*g* in a swinging-bucket rotor. The membranes at the interface between layers were collected by aspiration, diluted threefold with 20 mM HEPES pH 7.4 and 150 mM NaCl, and centrifuged for 30 min at 120,000*g*. Membranes were resuspended in 20 mM HEPES pH 7.4, 150 mM NaCl, 5% glycerol, and 2.5% SMA (Xiran SL30010 P20, Polyscope), and incubated 2 h with rotation at room temperature. Solubilized membrane nano-discs were cleared of debris by centrifugation for 30 min at 120,000*g.*

Membrane protein isolation. All procedures were performed at 4°C, and all solutions contained Complete-Ultra protease-inhibitor cocktail (Roche). Cell pellets were suspended in 20 mM Tris pH 7.4, incubated 30 min with rotation, and centrifuged for 10 min at 3,200*g*. Pellets were resuspended in 20 mM HEPES pH 7.4, 150 mM NaCl, 5% glycerol, and 1x Benzonase

(MilliporeSigma), and homogenized in a Dounce homogenizer. During homogenization, 1% n-Dodecyl-β-D-Maltopyranoside (DDM; Anatrace) was added and samples were incubated 2 h with rotation. Solubilized membrane-protein fractions were cleared of debris by centrifugation for 30 min at 180,000*g.*

Affinity purification of membrane proteins. Anti-1D4 resin was prepared by conjugating purified, anti-Rho antibody (1D4) to CNBr-activated sepharose 4B beads (Cytiva), following the manufacturer's protocol. The membrane-protein fraction from Expi293 cells expressing MFRP was mixed with anti-1D4 sepharose and incubated overnight at 4°C with gentle rotation. Beads were collected and washed 5 times (10 min each) with 20 mM HEPES pH 7.4, 150 mM NaCl, 5% glycerol, and 0.1% DDM. Next, protein bound to the 1D4 resin was subjected to detergent-amphipol exchange by adding 30 mg A8-35 (Anatrace) per 2 ml of 50% slurry and incubating for 1 h at 4°C with gentle rotation. Beads were washed 5 times (10 min each) with 20 mM HEPES pH 7.4, 150 mM NaCl, and 5% glycerol; then the protein was eluted with 2 washes of a solution of 1D4 peptide (2 mg/ml, Genscript) in 20 mM HEPES pH 7.4, 150 mM NaCl, and 5% glycerol, mixed each time for 1 h at 4°C by gentle rotation. Pooled eluates were concentrated with a 30 kDa MWCO Amicon Ultra-4 filter device (MilliporeSigma) at 4°C, and peptide was washed out by 3 subsequent washes with 20 mM HEPES pH 7.4, 150 mM NaCl, and 5% glycerol.

Secretory protein affinity purification. Anti-DYKDDDDK magnetic agarose (Thermo Fisher Scientific) was added to the culture-conditioned medium and incubated overnight at 4°C with gentle rotation. Beads were collected, washed 5 times (10 min each) with 20 mM HEPES pH 7.4, 150 mM NaCl, and 5% glycerol; then the protein was eluted with 2 washes, each with a solution of 1.5 mg/ml of 3x DYKDDDDK peptide (ApexBio) in 20 mM HEPES pH 7.4, 150 mM NaCl, 5% glycerol, mixed for 30 min at RT with gentle rotation. Pooled eluates were concentrated in a 10 kDa MWCO Amicon Ultra-15 filter device (MilliporeSigma) at 4°C; and peptide was washed out by 3 subsequent washes with 20 mM HEPES pH 7.4, 150 mM NaCl, 5% glycerol.

Affinity-purification mass spectrometry. Antibody conjugation to Dynabeads Protein-G magnetic beads (Thermo Fisher Scientific) was performed following the manufacturer's protocol, using the antibodies listed in Table S4. Beads were washed with 20 mM HEPES pH 7.4, 150 mM NaCl, and 5% glycerol, mixed with the SMA-solubilized bovine RPE membranes at a ratio of 3 mg total protein per 30 μg preabsorbed antibody per sample, and incubated with rotation, overnight at 4°C. The presence of MFRP in the samples was confirmed by immunoblotting with an antibody different from the one used for purification (Table S4). Beads were further washed three times with 20 mM HEPES pH 7.4, 150 mM NaCl, 5% glycerol, and six times with 50 mM

triethylammonium bicarbonate buffer pH 8.5 (TEAB; MilliporeSigma); then suspended in TEAB. Roughly 2 ug of trypsin was added for a 2-h digestion at 37°C, followed by another addition of about the same amount of trypsin before overnight incubation. After centrifugation at ----*g* for – min at RT, the supernatant with peptides was lyophilized and re-constituted in water containing 2% acetonitrile (ACN). LC-MS analysis was performed on a nanoElute UHPLC system with a ZDV spray emitter (Bruker). Peptides were separated at 40°C on a PepSep C18 column (1.5 µm particle size, 100 Å pores, 150 µm x 25 cm; Bruker) in a constant flow of 400 nL/min, using mobile phases A and B: water with 0.1% formic acid (v/v) (A), and 80/20/0.1% ACN/water/formic acid (v/v/v) (B). Peptides were separated using the following gradient: 0-60 min linear gradient of 5 to 27.5% B, 60-90 min linear increase to 37.5% B, 90-100 min linear increase to 55% B, followed by a washing step with 95% B, then re-equilibration. Peptides were eluted directly into the MS instrument: timsTOF Pro hybrid trapped ion mobility spectrometry-quadrupole time of flight MS with a modified CaptiveSpray nano-electrospray ion source (Bruker). The instrument was operated in the parallel accumulation serial fragmentation mode. Mass spectra were acquired *via* data-independent analysis (DIA), which consisted of four 25 m/z precursor windows per 100 ms TIMS scan. Sixteen TIMS scans, creating 64 total windows, layered the doubly and triply charged peptides on the m/z and ion mobility plane. Precursor windows began at 400 m/z and continued to 1200 m/z. The collision energy was ramped down linearly as a function of the mobility from 63 eV at 1/K0=1.5 Vs cm^{-2} to 17 eV at 1/K0=0.55 Vs cm^{-2}. Data were processed with Spectronaut v.17 (Biognosys), using the DirectDIA analysis mode with default mass tolerance/accuracy for precursor and fragment identification settings. The UniProt database of reviewed *Bos taurus* proteins, and a database of 112 common laboratory contaminants (https://www.thegpm.org/crap/) were used. A maximum of two missing cleavages were allowed, the required minimum peptide sequence length was 7 amino acids, and the peptide mass was limited to a maximum of 4600 Da. Carbamidomethylation of Cys residues was set as a fixed modification, and Met oxidation and acetylation of protein N termini were variable modifications. A decoy false-discovery rate (FDR) at less than 1% for peptide-spectrum matches and protein-group identifications was used for spectrum filtering. Decoy database hits, proteins identified as potential contaminants, and proteins identified exclusively by one site modification were excluded from further analysis. The MS proteomics data have been deposited to the ProteomeXchange Consortium *via* the MassIVE partner repository; the data set identifier is MSV000096610.

Immunoblotting. For mouse eyes, the neural retina and posterior eyecup (RPE, choroid, sclera) were separated by dissection. Tissue samples pooled from one animal were incubated for 30 min at 4°C with shaking in 50 mM Tris, pH 7.4, 150 mM NaCl, 1% sodium dodecyl sulfate (SDS), 5

mM tris-(2-carboxyethyl)phosphine, supplemented with Complete Ultra protease inhibitor cocktail (Roche). Samples were further sonicated for 1 min on ice and centrifuged at 21,000*g* for 15 min at 4°C. Supernatants were mixed with 4x Laemmli Sample Buffer (Bio-Rad). For recombinant protein detection, 4x Laemmli Sample Buffer was used also. Samples were separated on 4-20% polyacrylamide gradient gels (Bio-Rad) and transferred onto 0.2-μm nitrocellulose membranes (Bio-Rad), using an eBlot L1 wet-transfer system (Genscript), according to the manufacturer's recommendations. Membranes were incubated in the blocking buffer: 5% (w/v) nonfat milk (Research Products International) in Tris-buffered saline (TBS) with 0.1% Tween-20 (TBS-T) for 1 h at room temperature (RT); and subsequently incubated overnight at 4°C in the blocking buffer with the appropriate primary antibodies, as listed in Table S4. After 3 washes with TBS-T, membranes were incubated in blocking buffer with secondary antibodies (Table S4) for 1 h at RT, and subjected to three more washes with TBS-T. For detection of the horseradish peroxidase (HRP)-conjugated antibody, SuperSignal West Pico-Plus Chemiluminescent Substrate (Thermo Fisher Scientific) was used. Band visualization was performed using the Odyssey (Li-Cor) or ChemiDoc (Bio-Rad) imaging systems.

Lipid binding assay using dot blots. Membrane lipid strips (Echelon Biosciences) were washed in 20 mM HEPES pH 7.4, 150 mM NaCl, 0.1% Tween-20 (MilliporeSigma) for 1 h at RT and blocked in 20 mM HEPES pH 7.4, 150 mM NaCl, 0.1% Tween-20, 3% FA-free BSA overnight at 4°C, with gentle agitation. 50 μg of purified recombinant protein in blocking buffer was added per dot blot and incubated for 8 h at RT. Strips were subsequently washed 3 times (5 min each) in wash buffer; then incubated for 2 h in the blocking buffer with the appropriate primary antibodies, as listed in Table S4. After 3 washes with the wash buffer, the samples were incubated for 1 h at RT in blocking buffer with HRP-conjugated secondary antibodies (Table S4), followed by a final three washes in wash buffer. Chemiluminescence images were acquired using SuperSignal West Pico-Plus Chemiluminescent Substrate and the ChemiDoc imaging system.

Lipid binding assay on resins. Lipid resins were prepared by coupling biotinylated-PS or -PC (Echelon Biosciences) with T1 streptavidin-coated magnetic beads (Dynabeads, Thermo Fisher Scientific), following the manufacturers recommendations. Coupled or uncoupled (control) beads were added to the membrane protein fractions from Expi293 cells expressing MFRP-1D4, solubilized with 1% DDM, and incubated with mild agitation for 18 h at 4°C. Resins were subsequently washed 5 times (10 min each) with 20 mM HEPES pH 7.4, 150 mM NaCl, 5% glycerol, and 0.1% DDM; then resuspended in Laemmli sample buffer (Bio-Rad) to release the proteins. Proteins bound to, then released from the resins were analyzed by immunoblotting.

Protein binding assay on resins. Anti-1D4 and anti-FLAG resins were prepared by conjugating purified, anti-Rho (1D4) or anti-FLAG antibody (Table S4) with Dynabeads Protein-G magnetic beads following the manufacturer's protocol. Beads were washed with 20 mM HEPES pH 7.4, 150 mM NaCl, 5% glycerol, and 0.1% DDM. Next, the membrane-protein fraction from Expi293 cells expressing the bait protein was mixed with the appropriate resin and incubated for 4 h at 4°C with gentle rotation. Beads were collected and washed 5 times (10 min each) with 20 mM HEPES pH 7.4, 150 mM NaCl, 5% glycerol, and 0.1% DDM. Next, the membrane-protein fraction from Expi293 cells expressing the prey protein was added and incubated for 18 h at 4°C with gentle rotation. Resins were subsequently washed 5 times (10 min each) with 20 mM HEPES pH 7.4, 150 mM NaCl, 5% glycerol, and 0.1% DDM; then resuspended in Laemmli sample buffer (Bio-Rad) to release the proteins. Proteins bound to, then released from the resins were analyzed by immunoblotting.

Transmission Electron Microscopy. Enucleated mouse eyes were dissected in PBS, and the resulting eyecups were processed as described previously (24), with the following modification. After 3-4 h fixation at room temperature, eyecups were cut in halves and further fixed for 2 h at 33 °C. Plastic blocks were cut with a UCT Ultracut ultra-microtome (Leica Biosystems) into 80-90 nm sections, and picked up with 50 mesh nickel grids (Electron Microscopy Sciences) coated with handmade formvar film. After drying, sections were stained with the supernatant of 8% uranyl acetate in 50% ethanol for 10 minutes at room temperature, then rinsed with 50% ethanol. Samples were imaged with a Tecnai Spirit electron microscope (Thermo Fisher Scientific) at 80 kV at the Indiana University School of Medicine Center for Electron Microscopy.

Subretinal injections. Mice were anesthetized with an IP injection of a mixture of ketamine/xylazine solution (100/8.75 mg/kg) and their pupils were dilated with a drop of 1% tropicamide followed by 10%-phenylephrine ophthalmic solution (MWI Animal Health). Subretinal injections were performed with direct visualization under an ophthalmic surgical microscope (Zeiss). Corneas were hydrated with the application of GenTeal Severe-Lubricant Eye Gel (0.3% hypromellose, Alcon). An incision was made using a 26-gauge needle through the cornea, at the nasal side, adjacent to the limbus. A 34-gauge blunt-end needle (World Precision Instruments) connected to an RPE-KIT (World Precision Instruments) by SilFlex tubing (World Precision Instruments) was inserted through the corneal incision and advanced through the retina, avoiding the lens. Each mouse received a 1.5-µl injection per eye, composed of the lentivirus preparation (≥ 10^9 pfu/ml) or PBS (control) and AAV1-expressing EGFP (Addgene) mixed at a 4:1 ratio. Mice were wrapped in a 37°C heating pad for recovery, and anesthesia reversal was achieved with

intraperitoneal atipamezole (2.5 mg/kg; MWI Animal Health). Only mice that had minimal complications after subretinal injection were kept for further evaluation.

Figures S1 to S5

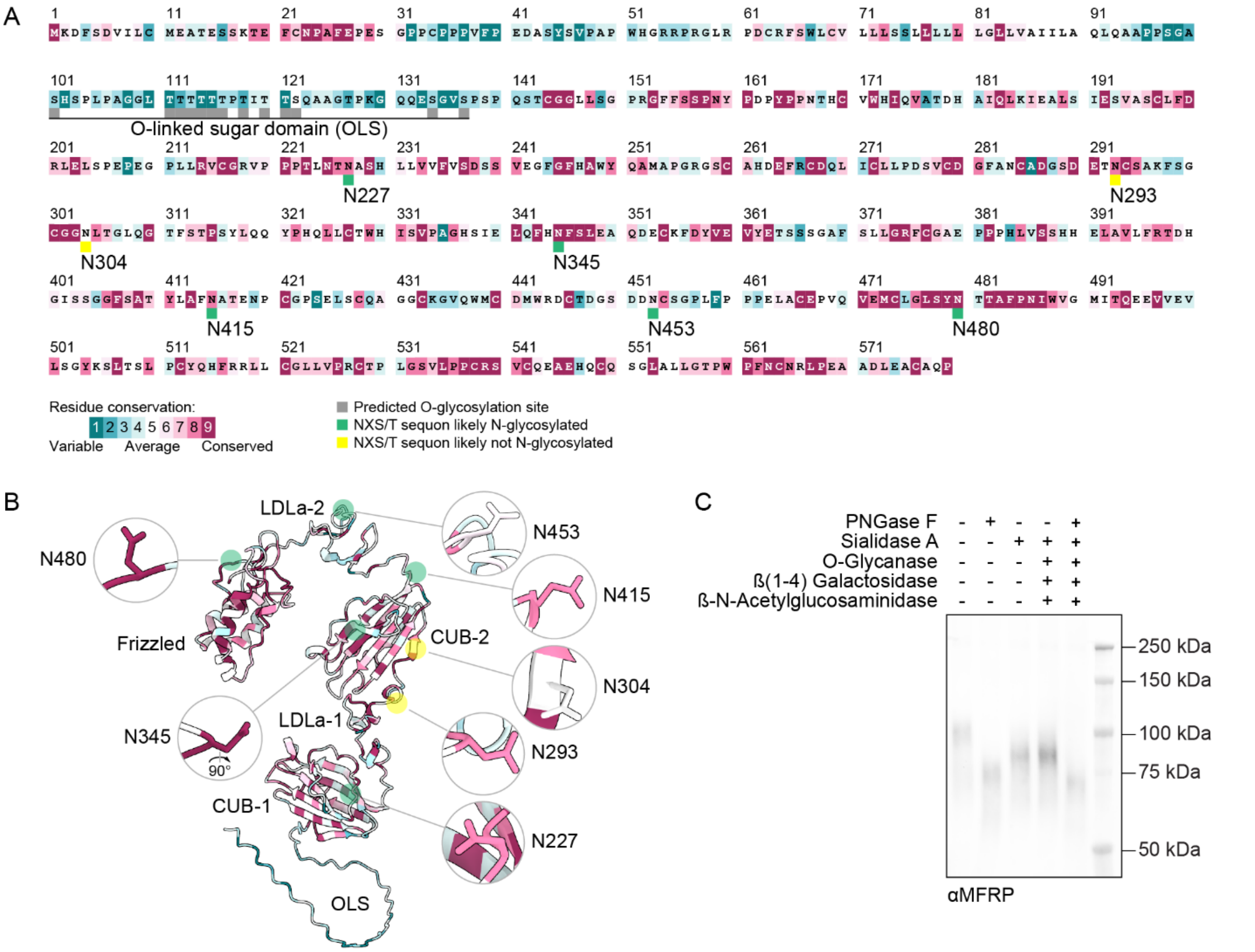

Figure S1. MFRP glycosylation status. A. Amino-acid sequence of human MFRP, with evolutionary conservation scores for each residue indicated by gradations in color. Specific residues are identified as sites of predicted O-glycosylation within the OLS domain, or potential N-glycosylation sites within the conserved NXS/T sequons. **B.** AlphaFold structural model of the extracellular portion of human MFRP, highlighting the potential N-glycosylation sites. **C.** Deglycosylation analysis of native bovine MFRP in SMA-solubilized RPE membranes, visualized by anti-MFRP immunoblotting.

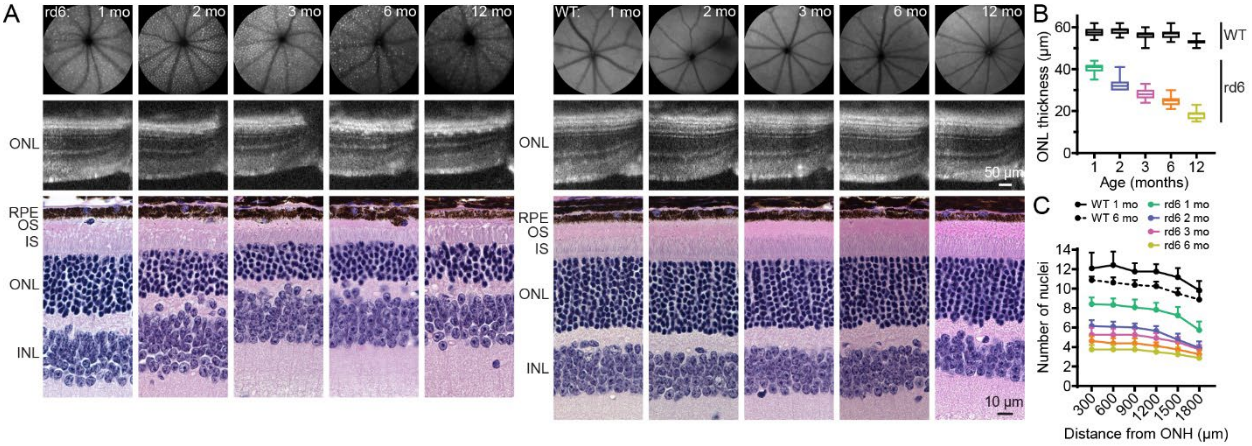

Figure S2. Structural characteristics of the retinas from rd6 mice. **A.** SLO images (top row, scale bars: 50 μm), retinal OCT images (middle row, scale bars: 50 μm), and H&E-stained retinal section (bottom row, scale bars: 10 μm) from 1-, 2-, 3-, 6- and 12-mo rd6 and WT animals. **B.** OCT-based quantification of ONL thickness in rd6 and WT mice of different ages, measured 500 μm from the ONH; n = 5 eyes (1-, 3-, 6-mo); n = 3 eyes (12-mo). Boxes indicate 25th- to 75th-percentile range with median depicted by the lines inside the boxes; whiskers indicate the min-to-max range. **C.** Number of nuclei per row quantified in histological sections of eyes from rd6 and WT mice of different ages. Data are shown as mean ± SEM; n = 12 eyes (1-, 3-mo), n = 8 eyes (6-, 12-mo).

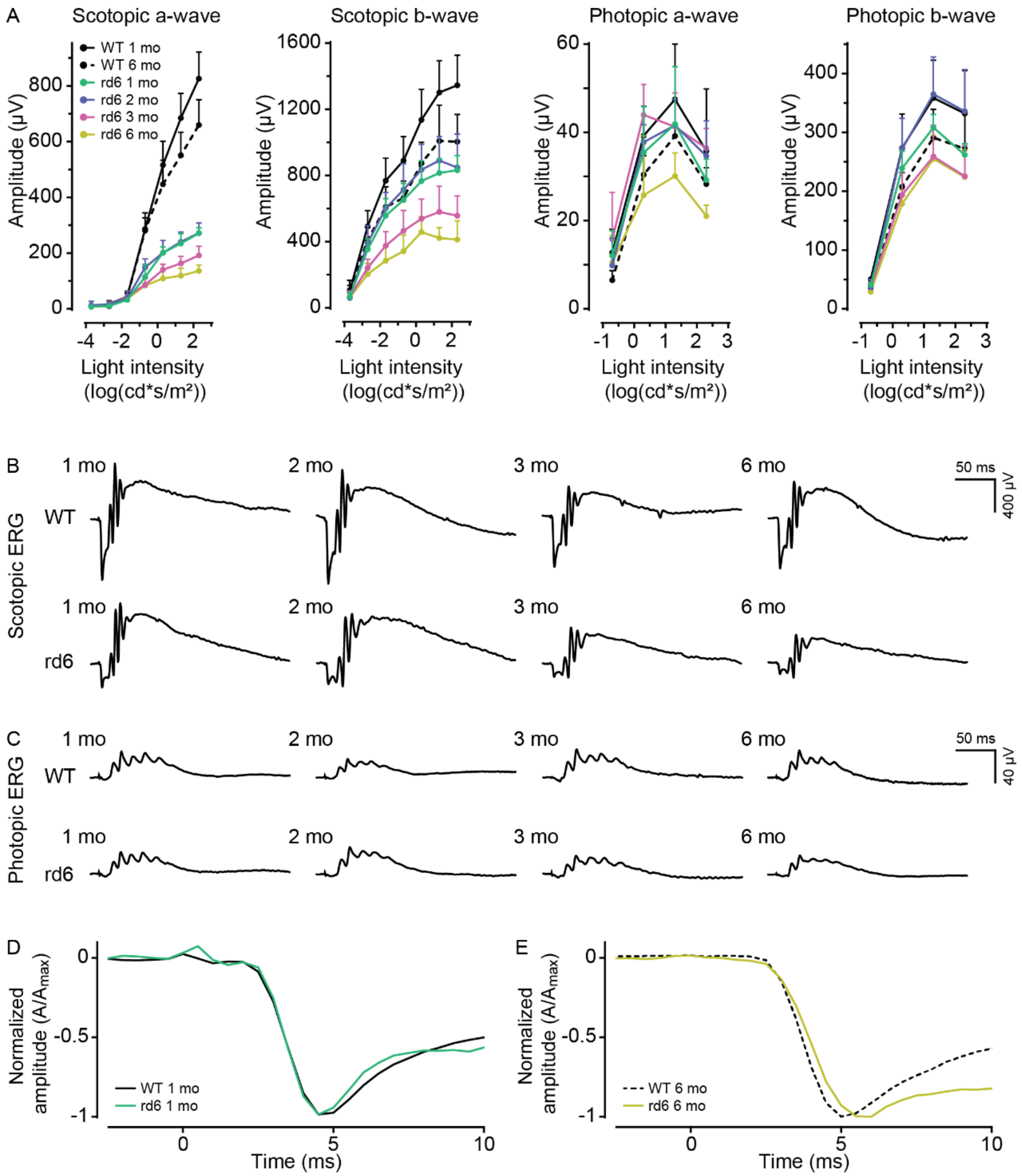

Figure S3. Functional characteristics of the retinas from rd6 mice. A. Amplitudes of serial ERG responses to increasing flash stimuli, obtained for selected light intensities under scotopic and photopic conditions with 1-, 2-, 3-, and 6-mo rd6 mice, and 1- and 6-mo WT mice. Values are plotted as mean ± SEM (top half of error bars shown); n = 5 eyes. **B-C.** Representative ERG waveforms of scotopic (B) and photopic (C) responses to a 2.3 log (cd·s/m^2) light stimulus. **D-E**. Comparison of the leading edge of the scotopic a-wave of 1-mo (D) and 6-mo (E) mice (from waveforms shown in B). For each mouse, the amplitude was normalized to the a-wave trough (A_{max}).

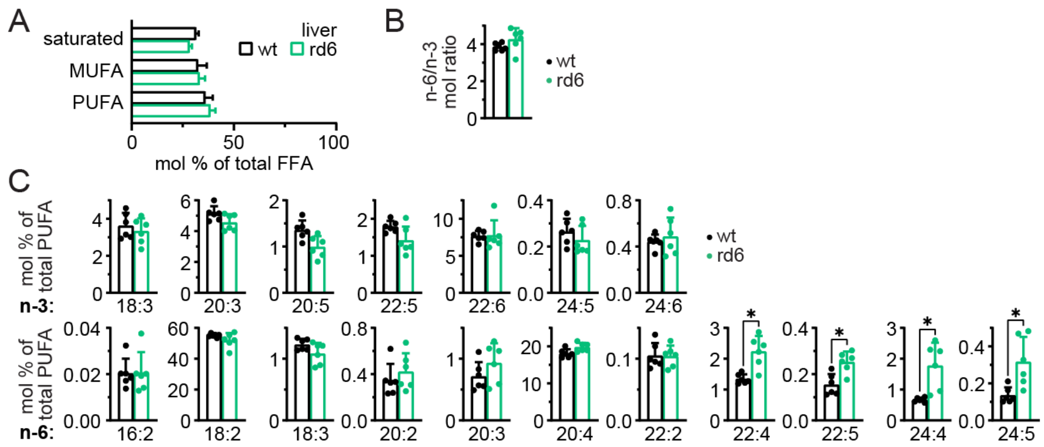

Figure S4. Analysis of PUFA metabolism in the MFRP-deficient liver. A. Lipidomic analysis of the FFA content in the liver of 1-mo rd6 and WT mice, divided into classes: saturated FA, MUFA, and PUFA. Each bar represents the mean ± SD of n = 6 independent replicates. Significant differences were not observed, two-way ANOVA with Holm-Šídák post-hoc test. **B.** Changes in the liver PUFA fraction composition in the MFRP-deficient liver. The graph shows mol ratios (total free n-6/n-3 PUFA). **C.** Graphs depict the percent composition of each molecular species of PUFA in the total PUFA content. Each bar represents the mean ± SD of n = 6 independent replicates. Significant differences are indicated as follows: *P < 0.05, Mann-Whitney test with correction for multiple comparisons.

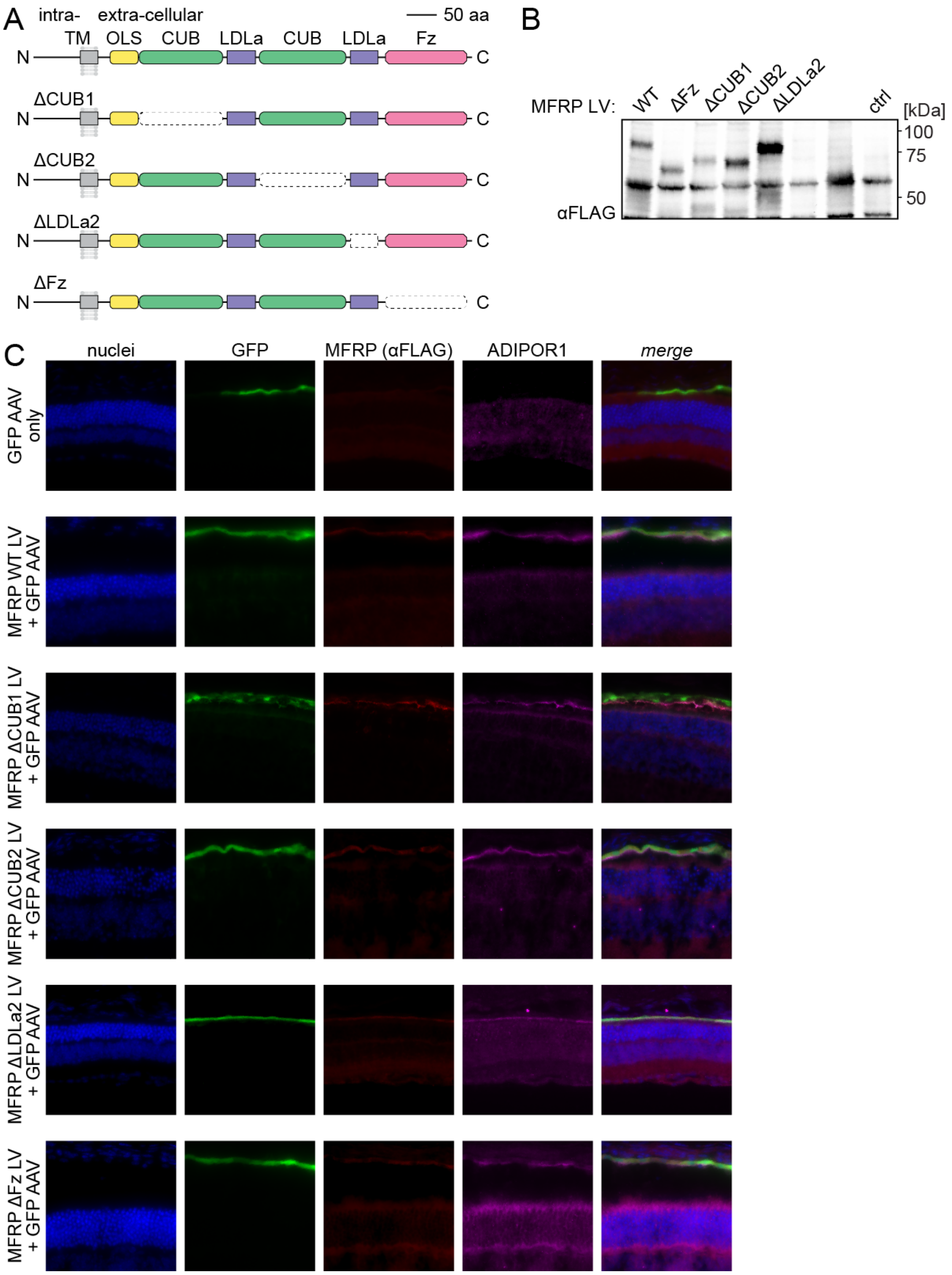

Figure S5. Analysis of the involvement of MFRP domains in the protein function. **A.** MFRP domain structure, and design of its single-domain deletion variants for functional studies. **B.** Expression of MFRP variants in HEK293 cells after lentiviral transfection. Lentiviruses encoded the respective single-domain-deletion MFRP variant, with C-terminal 3x FLAG epitope. **C.** IHC images of eye cryosections from *Mfrp*$^{rd6/rd6}$ animals on the albino (*Tyr*$^{c\text{-}2J/c\text{-}2J}$) background that were injected subretinally with lentivirus encoding the respective MFRP variant, along with a GFP-expressing AAV. Samples were stained to visualize MFRP, ADIPOR1 and nuclei.

Tables S1 to S5.

Table S1. Conserved sites for potential N-glycosylation predicted in the amino-acid sequences of human, bovine, and mouse MFRP by NetNglyc server (6). Listed for each site are the computed glycosylation potential (min. 0, max. 1, threshold 0.5), the number of networks supporting the prediction (jury agreement), and the overall prediction result.

Human					Bovine					Mouse				
**Posi-tion**	**Motif**	**Potential**	**Jury agree-ment**	**N-Glyc result**	**Posi-tion**	**Motif**	**Potential**	**Jury agree-ment**	**N-Glyc result**	**Posi-tion**	**Motif**	**Potential**	**Jury agree-ment**	**N-Glyc result**
227	NASH	0.6213	(9/9)	++	229	NASR	0.6686	(8/9)	+	233	NTSH	0.6428	(9/9)	++
293	NCSA	0.4621	(7/9)	-	295	NCSA	0.4236	(8/9)	-	299	NCSA	0.3943	(8/9)	-
304	NLTG	0.4994	(5/9)	-	306	NLTG	0.5182	(5/9)	+	310	NLTG	0.4753	(5/9)	-
345	NFSL	0.6433	(7/9)	+	347	NFSL	0.6884	(9/9)	++	351	NFSL	0.6483	(7/9)	+
415	NATE	0.6807	(9/9)	++	417	NATE	0.7004	(9/9)	++	421	NTTE	0.6253	(9/9)	++
453	NCSG	0.5046	(5/9)	+	452	NCSS	0.5821	(7/9)	+	458	NCSS	0.6063	(9/9)	++
480	NTTA	0.5927	(7/9)	+	479	NTTA	0.5594	(7/9)	+	485	NTTA	0.5923	(7/9)	+

Table S2. Potential O-glycosylation sites predicted in the amino-acid sequences of human, bovine, and mouse MFRP by NetOglyc server. Listed for each site are the computed glycosylation potential (min. 0, max. 1, threshold 0.5), and the overall prediction result. All positions with a potential greater than 0.5 are listed.

Human			Bovine			Mouse		
**Position**	**Potential**	**O-Glyc result**	**Position**	**Potential**	**O-Glyc result**	**Position**	**Potential**	**O-Glyc result**
S101	0.764	+	T95	0.883	+	S96	0.500	+
T111	0.697	+	T96	0.816	+	T100	0.541	+
T112	0.640	+	S97	0.904	+	T101	0.592	+
T113	0.713	+	T101	0.910	+	S118	0.776	+
T114	0.868	+	S107	0.755	+	T119	0.736	+
T115	0.797	+	T112	0.851	+	T120	0.786	+
T116	0.709	+	T113	0.894	+	T123	0.723	+
T118	0.615	+	T115	0.711	+	T124	0.866	+
T120	0.851	+	T116	0.890	+	T125	0.860	+
T121	0.802	+	T119	0.792	+	T126	0.872	+
S122	0.867	+	S120	0.934	+	T127	0.939	+
S134	0.691	+	T121	0.864	+	T128	0.940	+
S137	0.802	+	T122	0.868	+	T129	0.957	+
			T123	0.829	+	T130	0.953	+
			S124	0.778	+	T131	0.932	+
			T127	0.784	+	T135	0.908	+
			T129	0.591	+	S143	0.510	+
			S139	0.678	+			

Table S3. List of significantly up-regulated or down-regulated genes in the RPE of rd6 mice compared to control, exhibiting prominent expression in the RPE and involvement in lipid metabolism. Genes were selected based on the following criteria: average relative expression level ≥ 50% of Gapdh, ≥ 1.75-fold change (FC), adjusted P-value $q < 0.05$, association with ≥ 1 GO term or KEGG pathway related to fatty acid or lipid process.

Gene symbol	Relative expression	log2 (FC)	Relevant GO terms	Relevant KEGG pathways
**Upregulated in rd6**				
Cox8b	11827.3	2.4		non-alcoholic fatty liver disease
Hexb	3706.0	1.66	phospholipid biosynthetic process, lipid storage	glycosphingolipid biosynthesis
Irs1	783.3	1.64	regulation of fatty acid beta-oxidation	non-alcoholic fatty liver disease
Plin4	1723.6	1.45	lipid particle	
Id2	7371.5	1.35	regulation of lipid metabolic process	
Thbs1	53689.5	1.22	regulation of plasma membrane long-chain fatty acid transport	
Lpl	784.2	1.16	fatty acid biosynthetic process	glycerolipid metabolism
Stat3	3479.1	1.07	phospholipid homeostasis	
Aqp1	1909.3	0.92	lipid digestion	
Tnfrsf1a	3668.1	0.84		sphingolipid signaling pathway, non-alcoholic fatty liver disease
Adgrf5	1158.2	0.83	regulation of phospholipid biosynthetic process	
Arl6	980.4	0.80	phospholipid binding	
**Downregulated in rd6**				
Plbd1	1767.1	-0.83	lipid metabolic process	
Akt3	2031.6	-0.84		sphingolipid signaling pathway, non-alcoholic fatty liver disease
Fads2	5874.5	-0.85	unsaturated fatty acid biosynthetic process	biosynthesis of unsaturated fatty acids
Sec14l2	1938.0	-0.87	lipid binding	
Acsl6	35522.7	-0.89	fatty acid transport; phospholipid biosynthetic process	fatty acid biosynthesis, fatty acid degradation
Inpp4b	1720.2	-0.90	lipid binding	phosphatidylinositol signaling system
Slc16a1	37050.1	-0.92	lipid metabolic process	
Myo5a	2802.7	-0.95	long-chain fatty acid biosynthetic process	
Lrat	72435.8	-0.98	regulation of lipid transport	
Ptges	5620.4	-0.99	fatty acid biosynthetic process	

Irs2	4576.4	-1.05	regulation of plasma membrane long-chain fatty acid transport, regulation of fatty acid beta-oxidation	non-alcoholic fatty liver disease
Elovl2	1544.5	-1.07	fatty acid biosynthetic process, fatty acid elongation	biosynthesis of unsaturated fatty acids, fatty acid elongation
Enpp2	132879.0	-1.32	phospholipid catabolic process	ether lipid metabolism
Acot1	1077.2	-1.39	long-chain fatty acid metabolic process	biosynthesis of unsaturated fatty acids, fatty acid elongation
Angptl4	1419.4	-1.85	regulation of lipid metabolic process	
Apod	1177.6	-1.97	regulation of lipoprotein lipid oxidation	
Mfsd2a	1212.2	-2.03	lipid transport across blood brain barrier, fatty acid transport	

Table S4. List of antibodies used in the study [a].

Target	Host	Source	Identifier	Application and dilution
**Primary antibodies**				
1D4 (TETSQVAPA)	mouse	Produced in-house	1D4	IP, DB 0.1 µg/ml
1D4 (TETSQVAPA) AF647	mouse	Produced in-house	1D4	WB 0.1 µg/ml
ADIPOR1 (mouse)	rabbit	IBL America	18993	IHC 1:100; WB 1:500
FLAG (DYKDDDDK)	mouse	MilliporeSigma	F1804	DB, WB 1:1000
FLAG (DYKDDDDK) Bio	mouse	MilliporeSigma	F9291	IHC 1:100
FLAG (DYKDDDDK)	rat	Thermo Fisher Scientific	MA1-142	IP
GAPDH (mouse)	goat	R&D Systems	10494-1-AP	WB 1:1000
GLUT1 (mouse)	rabbit	Thermo Fisher Scientific	MA5-31960	IHC 1:100
KCNJ13 (mouse)	rabbit	Alomone Labs	APC-125	WB 1:1000
KCNJ13 (mouse)	rabbit	Santa Cruz Biotechnology	sc-398810	IHC 1:100
MFRP (bovine, human)	goat	R&D Systems	AF1915	IP; IHC 1:100; WB 1:500
MFRP (bovine)	mouse	Abnova	H00083552-M02	WB 1:500
MFRP (mouse)	goat	R&D Systems	AF3445	IHC 1:100; WB 1:500
ZO-1 (mouse)	rabbit	Thermo Fisher Scientific	61-7300	IHC 1:100
Normal IgG	goat	R&D Systems	AB-108-C	IP
Normal IgG	mouse	Santa Cruz Biotechnology	sc-2025	IP
Normal IgG	rat	R&D Systems	6-001-A	IP
**Secondary antibodies**				
Goat IgG AF647	donkey	Thermo Fisher Scientific	A32849	IHC 1:500
Goat IgG HRP	donkey	Thermo Fisher Scientific	A16005	WB 1:10000
Mouse IgG HRP	goat	Promega	W4021	DB, WB 1:2500
Rabbit IgG AF488	donkey	Thermo Fisher Scientific	A21206	IHC 1:500
Rabbit IgG HRP	donkey	Thermo Fisher Scientific	A16035	WB 1:10000

[a] Abbreviations used: AF, Alexa Fluor; IgG, Immunoglobulin G; DB, Dot blot; IHC, Immunohistochemistry; IP, Immunoprecipitation; WB, Western blot.

Table S5. List of DNA primers used in the study.

Target	Oligo sequence
ELOVL2 (F)	ACCACGCCTCCATGTTTAAC
ELOVL2 (R)	TGGGGCCAAAGAAACTTTGC
ELOVL4 (F)	TGTTGCAGCTGGTTCAGTTC
ELOVL4 (R)	ATAGACAATGAGAGCCCAGTGC
FADS2 (F)	TCACTTAAAGGGTGCCTCTGC
FADS2 (R)	AACACGTGCAGCATGTTCAC
GAPDH (F)	ATGCCCCCATGTTTGTGATG
GAPDH (R)	AGGAGGCATTGCTGACAATC

SI References.

1. B. J. Raney, *et al.*, The UCSC Genome Browser database: 2024 update. *Nucleic Acids Res.* **52**, D1082–D1088 (2024).
2. F. Aguet, *et al.*, The GTEx Consortium atlas of genetic regulatory effects across human tissues. *Science (80-.).* **369**, 1318–1330 (2020).
3. F. Abascal, *et al.*, Expanded encyclopaedias of DNA elements in the human and mouse genomes. *Nature* **583**, 699–710 (2020).
4. J. P. Ling, *et al.*, ASCOT identifies key regulators of neuronal subtype-specific splicing. *Nat. Commun. 2020 111* **11**, 1–12 (2020).
5. T. Paysan-Lafosse, *et al.*, InterPro in 2022. *Nucleic Acids Res.* **51**, D418–D427 (2023).
6. R. Gupta, S. Brunak, Prediction of glycosylation across the human proteome and the correlation to protein function. *Pac. Symp. Biocomput.*, 310–22 (2002).
7. C. Steentoft, *et al.*, Precision mapping of the human O-GalNAc glycoproteome through SimpleCell technology. *EMBO J.* **32**, 1478–1488 (2013).
8. L. Tarhan, *et al.*, Single Cell Portal: an interactive home for single-cell genomics data. *bioRxiv*, 2023.07.13.548886 (2023).
9. A. Monavarfeshani, *et al.*, Transcriptomic analysis of the ocular posterior segment completes a cell atlas of the human eye. *Proc. Natl. Acad. Sci. U. S. A.* **120**, e2306153120 (2023).
10. M. Varadi, *et al.*, AlphaFold Protein Structure Database: massively expanding the structural coverage of protein-sequence space with high-accuracy models. *Nucleic Acids Res.* **50**, D439–D444 (2022).
11. E. C. Meng, *et al.*, UCSF ChimeraX: Tools for structure building and analysis. *Protein Sci.* **32**, e4792 (2023).
12. P. Bankhead, *et al.*, QuPath: Open source software for digital pathology image analysis. *Sci. Rep.* (2017) https:/doi.org/10.1038/s41598-017-17204-5.
13. C. Xin-Zhao Wang, K. Zhang, B. Aredo, H. Lu, R. L. Ufret-Vincenty, Novel method for the rapid isolation of RPE cells specifically for RNA extraction and analysis. *Exp. Eye Res.* **102**, 1–9 (2012).

14. Y. Liao, G. K. Smyth, W. Shi, The R package Rsubread is easier, faster, cheaper and better for alignment and quantification of RNA sequencing reads. *Nucleic Acids Res.* (2019) https:/doi.org/10.1093/nar/gkz114.

15. M. I. Love, W. Huber, S. Anders, Moderated estimation of fold change and dispersion for RNA-seq data with DESeq2. *Genome Biol.* (2014) https:/doi.org/10.1186/s13059-014-0550-8.

16. A. Subramanian, *et al.*, Gene set enrichment analysis: A knowledge-based approach for interpreting genome-wide expression profiles. *Proc. Natl. Acad. Sci. U. S. A.* (2005) https:/doi.org/10.1073/pnas.0506580102.

17. B. Jassal, *et al.*, The reactome pathway knowledgebase. *Nucleic Acids Res.* **48**, D498–D503 (2020).

18. M. Kanehisa, Y. Sato, M. Furumichi, K. Morishima, M. Tanabe, New approach for understanding genome variations in KEGG. *Nucleic Acids Res.* (2019) https:/doi.org/10.1093/nar/gky962.

19. S. Carbon, *et al.*, The Gene Ontology Resource: 20 years and still GOing strong. *Nucleic Acids Res.* (2019) https:/doi.org/10.1093/nar/gky1055.

20. E. H. Choi, *et al.*, Insights into the pathogenesis of dominant retinitis pigmentosa associated with a D477G mutation in RPE65. *Hum. Mol. Genet.* (2018) https:/doi.org/10.1093/hmg/ddy128.

21. E. G. BLIGH, W. J. DYER, A rapid method of total lipid extraction and purification. *Can. J. Biochem. Physiol.* **37**, 911–917 (1959).

22. B. MacLean, *et al.*, Skyline: An open source document editor for creating and analyzing targeted proteomics experiments. *Bioinformatics* **26**, 966–968 (2010).

23. J. C. Saari, D. L. Bredberg, Acyl-CoA:Retinol acyltransferase and lecithin:Retinol acyltransferase activities of bovine retinal pigment epithelial microsomes. *Methods Enzymol.* **190**, 156–163 (1990).

24. S. Sakami, A. V. Kolesnikov, V. J. Kefalov, K. Palczewski, P23H opsin knock-in mice reveal a novel step in retinal rod disc morphogenesis. *Hum. Mol. Genet.* **23**, 1723–1741 (2014).
